# Estimation and Inference for High Dimensional Generalized Linear Models: A Splitting and Smoothing Approach

**Published:** 2021

**Authors:** Zhe Fei, Yi Li

**Affiliations:** Department of Biostatistics, UCLA, Los Angeles, California, 90025; Department of Biostatistics, University of Michigan, Ann Arbor, Michigan, 48109

**Keywords:** Confidence intervals, dimension reduction, high dimensional inference for GLMs, sparsity, sure screening

## Abstract

The focus of modern biomedical studies has gradually shifted to explanation and estimation of joint effects of high dimensional predictors on disease risks. Quantifying uncertainty in these estimates may provide valuable insight into prevention strategies or treatment decisions for both patients and physicians. High dimensional inference, including confidence intervals and hypothesis testing, has sparked much interest. While much work has been done in the linear regression setting, there is lack of literature on inference for high dimensional generalized linear models. We propose a novel and computationally feasible method, which accommodates a variety of outcome types, including normal, binomial, and Poisson data. We use a “splitting and smoothing” approach, which splits samples into two parts, performs variable selection using one part and conducts partial regression with the other part. Averaging the estimates over multiple random splits, we obtain the smoothed estimates, which are numerically stable. We show that the estimates are consistent, asymptotically normal, and construct confidence intervals with proper coverage probabilities for all predictors. We examine the finite sample performance of our method by comparing it with the existing methods and applying it to analyze a lung cancer cohort study.

## 1. Introduction

In the big data era, high dimensional regression has been widely used to address questions arising from many scientific fields, ranging from genomics to sociology (Hastie et al., 2009; Fan and Lv, 2010). For example, modern biomedical research has gradually shifted to understanding joint effects of high dimensional predictors on disease outcomes (e.g. molecular biomarkers on the onset of lung cancer) (Vaske et al., 2010; Chen and Yan, 2014, among others). A motivating clinical study is the Boston Lung Cancer Survivor Cohort (BLCSC), one of the largest comprehensive lung cancer survivor cohorts, which investigates the molecular mechanisms underlying lung cancer (Christiani, 2017). Using a target gene approach (Moon et al., 2003; Garrigos et al., 2018; Ho et al., 2019), we analyzed a subset of 708 lung cancer patients and 751 controls, with 6,800 single nucleotide polymorphisms (SNPs) from 15 cancer related genes, in addition to demographic variables such as age, gender, race, education level, and smoking status. Our objective was to determine which covariates were predictive in distinguishing cases from controls. As smoking is known to play a significant role in the development of lung cancer, we were interested in estimating and testing the interaction between smoking status (never versus ever smoked) and each SNP, in addition to the main effect of the SNP. Quantifying uncertainty of the estimated effects helps inform prevention strategies or treatment decisions for patients and physicians (Minnier et al., 2011).

Considerable progress has been made in drawing inferences based on penalized linear models (Zhang and Zhang, 2014; Javanmard and Montanari, 2014; Bühlmann et al., 2014; Dezeure et al., 2015). While techniques for variable selection and estimation in high dimensional settings have been extended to generalized linear models (GLMs) and beyond (Van de Geer, 2008; Fan et al., 2009; Witten and Tibshirani, 2009), high dimensional inference in these settings is still at its infancy stage. For example, Bühlmann et al. (2014) generalized the de-sparsified LASSO to high dimensional GLMs, while Ning and Liu (2017) proposed a de-correlated score test for penalized M-estimators. In the presence of high dimensional control variables, Belloni et al. (2014, 2016) proposed a post-double selection procedure for estimation and inference of a single treatment effect and Lee et al. (2016) characterized the distribution of a post-LASSO-selection estimator conditional on the *selected variables*, but only for the linear regression.

However, the performance of these methods may depend heavily on tuning parameters, often chosen by computationally intensive cross-validation. Also, these methods may require inverting a *p* × *p* information matrix (where *p* is the number of predictors), or equivalently, estimating a *p* × *p* precision matrix, with extensive computation and stringent technical conditions. For example, the sparse precision matrix assumption may be violated in GLMs, resulting in biased estimates (Xia et al., 2020).

We propose a new approach for drawing inference with high dimensional GLMs. The idea is to randomly split the samples into two sub-samples (Meinshausen et al., 2009), use the first sub-sample to select a subset of important predictors and achieve dimension reduction, and use the remaining samples to parallelly fit low dimensional GLMs by appending each predictor to the selected set, one at a time, to obtain the estimated coefficient for each predictor, regardless of being selected or not. As with other methods for high dimensional regression (Zhang and Zhang, 2014; Javanmard and Montanari, 2014; Bühlmann et al., 2014), one key assumption is that the number of non-zero components of β* is small relative to the sample size, where β* are the true values underlying the parameter vector, ***β***, in a regression model. The sparsity condition is reasonable in some biomedical applications. For example, in the context of cancer genomics, it is likely that a certain type of cancer is related to only a handful of oncogenes and tumor suppressor genes (Lee and Muller, 2010; Goossens et al., 2015). Under this sparsity condition, we show that our proposed estimates are consistent and asymptotically normal. However, these estimates can be highly variable due to both the random splitting of data and the variation incurred through selection. To stabilize the estimation and account for the variation induced by variable selection, we repeat the random splitting a number of times and average the resulting estimates to obtain the smoothed estimates. These smoothed estimators are consistent and asymptotically normal, with improved efficiency.

Our approach, termed Splitting and Smoothing for GLM (SSGLM), aligns with multi-sample splitting (Meinshausen et al., 2009; Wang et al., 2020) and bagging (Bühlmann and Yu, 2002; Friedman and Hall, 2007; Efron, 2014), and differs from the existing methods based on penalized regression (Zhang and Zhang, 2014; Bühlmann et al., 2014; Ning and Liu, 2017; Javanmard and Montanari, 2018). The procedure has several novelties. First, it addresses the high dimensional estimation problem through the aggregation of low dimensional estimations and presents computational advantages over other existing methods. For example, de-biased methods (Bühlmann et al., 2014; Javanmard and Montanari, 2018) require well-estimated high dimensional precision matrices for proper inference (e.g. correct coverage probabilities), which is statistically and computationally challenging. Complicated procedures that involve choosing a large number of tuning parameters are needed to strike a balance between estimation accuracy and model complexity; see Bühlmann et al. (2014) and Javanmard and Montanari (2014). In contrast, our algorithm is more straightforward as it avoids estimating a high dimensional precision matrix by adopting a “split and select” strategy with minimal tuning. Second, we have derived the variance estimator using the infinitesimal jackknife method adapted to the splitting and smoothing procedure (Efron, 2014). This is free of parametric assumptions and leads to confidence intervals with correct coverage probabilities. Third, we have relaxed the stringent “selection consistency” assumption on variable selection, which is required in Fei et al. (2019). Our procedure is valid with a mild “sure screening” assumption for the selection method. Finally, our framework facilitates hypothesis testing and drawing inference on predetermined contrasts in the presence of high dimensional nuisance parameters.

The rest of the paper is organized as follows. Section 2 describes the SSGLM procedure and Section 3 introduces its theoretical properties. Section 4 describes the inferential procedure and Section 5 extends it to accommodate any sub-vectors of parameters of interest. Section 6 provides simulations and comparisons with the existing methods. Section 7 reports our analysis of the BLCSC data. We conclude the paper with a brief discussion in Section 8.

## 2. Method

### 2.1 Notation

We assume the observed data (*Y_i_*, **x**_*i*_) = (*Y_i_*, *x*_*i*1_, *x*_*i*2_, …, *x_ip_*), *i* = 1, …, *n*, are i.i.d. copies of (*Y*, **x**) = (*Y, x*_1_, *x*_2_, …, *x_p_*). Without loss of generality, we assume that the predictors are centered with **E**(*x_j_*) = 0, *j* = 1, …, *p*. In the matrix form, we denote the *n* samples of observed data by **D**^(*n*)^ = (**Y, X**), where **Y** = (*Y*_1_, …, *Y_n_*)^T^ and **X** = (**X**_1_, …, **X**_*p*_). Here, **X**_*j*_ = (*x*_1*j*_, …, *x_nj_*)^T^ for *j* = 1, …, *p*. In addition, $\bar{X}=(1,X)$ includes an *n* × 1 column vector of 1’s. To accommodate non-Gaussian outcomes, we assume the outcome variable belongs to the linear exponential distribution family, which includes the normal, Bernoulli, Poisson, and negative-binomial distributions. That is, given **x**, the conditional density function for *Y* is

(1) f(Y|θ)=exp{Yθ−A(θ)+c(Y)},

where *A*(·) is a specified function that links the mean of *Y* to **x** through *θ*. We assume the second derivative of *A*(*θ*) is continuous and positive. We consider the canonical mean parameter, $\theta =\bar{x}\beta$, where $\bar{x}=(1,x)$ and ***β*** = (*β*_0_, *β*_1_, …, *β_p_*)^T^ include an intercept term. Specifically, denote $\mu =E\left(Y|x\right)=A^{\prime }\left(\theta \right)=g^{-1}\left(\bar{x}\beta \right)$, and **V**(*Y*|**x**) = *A*″(*θ*) = *ν*(*μ*), where *g*(·) and *ν*(·) are the link and variance functions, respectively.

The forms of *A*(·), *g*(·), and *ν*(·) depend on the data type of *Y*. For example, with the outcome in BLCSC being a binary indicator of lung cancer, *A*(*θ*) = log (1 + *e^θ^*), $g\left(\mu \right)=\text{logit}\left(\mu \right)=\text{log}\left(\frac{\mu }{1-\mu }\right)$ and *ν*(*μ*) = *μ*(1 − *μ*), corresponding to the well known logistic regression. Based on (**Y**, **X**), the negative log-likelihood with model (1) is

$$\ell \left(\beta \right)=\ell \left(\beta ;Y,X\right)=\frac{1}{n}\overset{}{\underset{i=1}{\sum }}\left\{A\left(\theta_{i}\right)-Y_{i}\theta_{i}\right\}=\frac{1}{n}\overset{n}{\underset{i=1}{\sum }}\left\{A\left({\bar{x}}_{i}\beta \right)-Y_{i}\left({\bar{x}}_{i}\beta \right)\right\},$$

where $\theta_{i}=\bar{x_{i}}\beta$ and $\bar{x_{i}}=(1,x_{i1},x_{i2},\ldots ,x_{\mathrm{ip}})$. The score and the observed information are

$$U\left(\beta \right)=\frac{1}{n}{\bar{X}}^{T}\left\{A^{\prime }\left(\bar{X}\beta -Y\right)\right\}\;\text{and}\;\hat{I}\left(\beta \right)=\frac{1}{n}{\bar{X}}^{T}V\bar{X},$$

which are a (*p* + 1) × 1 vector and a (*p* + 1) × (*p* + 1) matrix, respectively. Here, **V** = diag{*ν*(*μ*_1_), …, *ν*(*μ_n_*)} and $\mu_{i}=g^{-1}\left(\bar{x_{i}}\beta \right)$ for *i* = 1, …, *n*. When a univariate function such as *A*′(·) is applied to a vector, it operates component-wise and returns a vector of values.

We add an index set, *S* ⊂ {1, 2, …, *p*}, to the subscripts of vectors and matrices to index subvectors **x**_*iS*_ = (*x_ij_*)_*j*∈*S*_ and ${\bar{\text{x}}}_{iS}=\left(1,{\text{x}}_{iS}\right)$, and submatrices **X**_*S*_ = (**X**_*j*_)_*j*∈*S*_ and ${\bar{X}}_{S}=\left(1,X_{S}\right)$. Moreover, we define *S*_+j_ = {*j*} ∪ *S* and *S*_−*j*_ = *S*\{*j*}. As a convention, let *S*_+0_ = *S*_−0_ = *S*, where “0” corresponds to the intercept.

We write ***β***_S_ = (*β*_0_, *β*_j_)_*j*∈*S*_, which always includes the intercept and is of length 1 + |*S*|. The negative log-likelihood for model (1) that regresses **Y** on **X**_*S*_ (termed the partial regression) is

(2) $$\ell_{S}\left(\beta_{S}\right)=\ell \left(\beta_{S};Y,X_{S}\right)=\frac{1}{n}\overset{n}{\underset{i=1}{\sum }}\left\{A\left({\bar{x}}_{iS}\beta_{S}\right)-Y_{i}{\bar{x}}_{iS}\beta_{S}\right\}.$$

Similarly, $U_{S}\left(\beta_{S}\right)=n^{-1}{\bar{X}}_{S}^{T}\left(A^{\prime }\left({\bar{X}}_{S}\beta_{S}\right)-Y\right)$ and ${\hat{I}}_{S}\left(\beta_{S}\right)=n^{-1}{\bar{X}}_{S}^{T}V_{S}{\bar{X}}_{S}$, where $V_{S}=\text{diag}\left\{A^{\prime \prime }\left({\bar{x}}_{1S}\beta_{S}\right),\ldots ,A^{\prime \prime }\left({\bar{x}}_{nS}\beta_{S}\right)\right\}$. Let the true values of ***β*** be $\beta^{*}=\left(\beta_{0}^{*},\beta_{1}^{*},\ldots ,\beta_{p}^{*}\right)$. Define the expected information as $I^{*}=E\left\{\hat{I}\left(\beta^{*}\right)\right\}$. Let $S^{*}=\left\{j\neq 0:\beta_{j}^{*}\neq 0\right\}$ denote the active set, and let *s*_0_ = |S*| be the number of nonzero and non-intercept elements in β*. When *S* ⊇ S*, define the “observed” *sub-information* by ${\hat{I}}_{S}={\hat{I}}_{S}(\beta_{S}^{*})$, and the “expected” sub-information by $I_{S}=E\left\{{\hat{I}}_{S}\right\}$. The latter is equal to the submatrix of I* with rows and columns indexed by *S*, which is denoted by $I_{S}^{*}$.

### 2.2 Proposed SSGLM estimator

Under model (1), we assume a sparsity condition that *s*_0_ is small relative to the sample size and will be detailed in Section 3. We randomly split the samples, **D**^(*n*)^, into two parts, **D**_1_ and **D**_2_, with sample sizes |**D**_1_| = *n*_1_, |**D**_2_| = *n*_2_, respectively, such that *n*_1_ + *n*_2_ = *n*. For example, we can consider an equal splitting with *n*_1_ = *n*_2_ = *n*/2. We apply a variable selection scheme, $S_{\lambda }$, where *λ* denotes the tuning parameters, to **D**_2_ to select a subset of important predictors *S* ⊂ {1, …, *p*}, with |*S*| < *n* for dimension reduction. Then using **D**_1_ = (**Y**^1^, **X**^1^), for each *j* = 1, 2, …, *p*, we fit a low dimensional GLM by regressing **Y**^1^ on $X_{S_{+j}}^{1}$, where *S*_+*j*_ = {*j*} ∪ *S*. Denote the maximum likelihood estimate (MLE) of each fitted model as ${\tilde{\beta }}_{S_{+j}}$, and define ${\tilde{\beta }}_{j}={\left({\tilde{\beta }}_{S_{+j}}\right)}_{j}$, the element of ${\tilde{\beta }}_{S_{+j}}$ corresponding to predictor **X**_*j*_. We denote by ${\tilde{\beta }}_{0}$ the estimator of the intercept from the model $Y^{1}\sim X_{S}^{1}$. Thus, the one-time estimator based on a single data split is defined as

(3) $${\tilde{\beta }}_{S_{+j}}=\underset{\beta_{S_{+j}}}{\text{argmin}}\;\ell_{S_{+j}}\left(\beta_{S_{+j}}\right)=\underset{\beta_{S_{+j}}}{\text{argmin}}\;\ell \left(\beta_{S_{+j}};Y^{1},X_{S_{+j}}^{1}\right);\;{\tilde{\beta }}_{j}={\left({\tilde{\beta }}_{S_{+j}}\right)}_{j};\;\tilde{\beta }\text{=}\left({\tilde{\beta }}_{0},{\tilde{\beta }}_{1},\ldots ,{\tilde{\beta }}_{p}\right).$$

In the linear regression setting (Fei et al., 2019), ${\tilde{\beta }}_{j}$ in (3) has an explicit form,β~j={(XS+j1XTS+j1)−1XS+j1YT1}j.

The rationale for this one-time estimator is that if the subset of important predictors, *S*, is equal to or contains the active set, S*, then ${\tilde{\beta }}_{j}$ would be a consistent estimator regardless of whether variable *j* is selected or not (Fei et al., 2019). We show in Theorem 1 that the one-time estimator is indeed consistent and asymptotically normal in the GLM setting.

However, the estimator based on a single split is highly variable, making it difficult to separate true signals from noises. This phenomenon is analogous to using a single tree in the bagging algorithm (Bühlmann and Yu, 2002). To reduce this variability, we resort to a multi-sample splitting scheme. We randomly split the data multiple times, repeat the estimation procedure, and average the resulting estimates to obtain the smoothed coefficient estimates. Specifically, for each *b* = 1, 2, …, *B*, where *B* is large, we randomly split the data, **D**^(*n*)^, into $D_{1}^{b}$ and $D_{2}^{b}$, with $|D_{1}^{b}|=n_{1}$ and $|D_{2}^{b}|=n_{2}$ such that the splitting proportion is *q* = *n*_1_/*n*, 0 < *q* < 1. Denote the candidate set of variables selected by applying $S_{\lambda }$ to $D_{2}^{b}$ as *S^b^*, and the estimates via (3), as ${\tilde{\beta }}^{b}=\left({\tilde{\beta }}_{0}^{b},{\tilde{\beta }}_{1}^{b},\ldots ,{\tilde{\beta }}_{p}^{b}\right)$. Then the smoothed estimator, termed the SSGLM estimator, is defined to be

(4) $$\hat{\beta }=\left({\hat{\beta }}_{0},{\hat{\beta }}_{1},\ldots ,{\hat{\beta }}_{p}\right),\;\mathrm{where}\;{\hat{\beta }}_{j}=\frac{1}{B}\overset{B}{\underset{b=1}{\sum }}{\tilde{\beta }}_{j}^{b}.$$

The procedure is described in Algorithm 1.

## 3. Theoretical Results

We specify the following regularity conditions.

(A1) (*Bounded observations*) ‖**x**‖_∞_ ≤ *C*_0_ and **E**|*Y*| < ∞. Without loss of generality, we assume *C*_0_ = 1.
(A2) (*Bounded eigenvalues and effects*) The eigenvalues of $\Sigma =E\left({\bar{x}}^{T}\bar{x}\right)$, where $\bar{x}=(1,x)$, are bounded below and above by constants *c*_min_, *c*_max_, such that
$$0<c_{\min}\leq \lambda_{\min}\left(\Sigma \right)<\lambda_{\max}\left(\Sigma \right)\leq c_{\max}<\infty .$$
  In addition, there exists a constant *c_β_* > 0 such that |β*|_∞_ ≤ *c_β_*.
(A3) (*Sparsity and sure screening property*) Recall that $S^{*}=\left\{j\neq 0:\beta_{j}^{*}\neq 0\right\}$ and $s_{0}=\left|S^{*}\right|$. Let ${\hat{S}}_{\lambda_{n}}$ be the index set of predictors selected by S with a tuning parameter *λ_η_*. Assume log *p* = *o*(*n*^1/2^), there exists a sequence {*λ_n_*}_*n*≥1_ and constants 0 < *c*_1_ < 1/2, *c*_2_, *K*_1_, *K*_2_ > 0 such that *s*_0_ ≤ *K*_1_*n*^*c*_1_^, $\left|{\hat{S}}_{\lambda_{n}}\right|\leq K_{1}n^{c_{1}}$, and
$$\text{P}\left(S^{*}\subseteq {\hat{S}}_{\lambda_{n}}\right)\geq 1-K_{2}{\left(p\vee n\right)}^{-1-c_{2}}.$$

**Algorithm 1:** SSGLM Estimator

**Require:** A variable selection procedure denoted by $S_{\lambda }$	
**Input:** Data (**Y**, **X**), a splitting proportion *q* ∈ (0, 1), and the number of random splits *B*	
**Output:** Coefficient vector estimator $\hat{\beta }$	
1:	**for** *b* = 1, 2, …, *B* **do**
2:	Split the samples into **D**_1_ and **D**_2_, with |**D**_1_| = *qn*, |**D**_2_| = (1 − *q*)*n*
3:	Apply $S_{\lambda }$ to **D**_2_ to select predictors indexed by *S* ⊂ {1, …, *p*}
4:	**for** *j* = 0, 1, …, *p* **do**
5:	With *S*_+*j*_ = {*j*} ∪ *S*, fit model (1) by regressing **Y**^1^ on $X_{S_{+j}}^{1}$, where **D**_1_ = (**Y**^1^, **X**^1^), and compute the MLE ${\tilde{\beta }}_{S_{+j}}$ as in (3)
6:	Compute ${\tilde{\beta }}_{j}^{b}={\left({\tilde{\beta }}_{S_{+j}^{b}}\right)}_{j}$, which is the coefficient for predictor **X**_*j*_ (${\tilde{\beta }}_{0}^{b}$ represents the intercept)
7:	**end for**
8:	Output ${\tilde{\beta }}^{b}=({\tilde{\beta }}_{0}^{b},{\tilde{\beta }}_{1}^{b},\ldots ,{\tilde{\beta }}_{p}^{b})$
9:	**end for**
10:	Compute $\hat{\beta }=({\hat{\beta }}_{0},{\hat{\beta }}_{1},\ldots ,{\hat{\beta }}_{p})$, where ${\hat{\beta }}_{j}=\frac{1}{B}\sum_{b=1}^{B}{\tilde{\beta }}_{j}^{b}$

**Algorithm 2:** Model-free Variance Estimator

**Input:** *n, n*_1_, *B*, ${\tilde{\beta }}^{b}$, *b* = 1, 2, …, *B* and $\hat{\beta }$	
**Output:** Variance estimator ${\hat{V}}_{j}^{B}$ for ${\hat{\beta }}_{j}$, *j* = 0, 1, …, *p*	
1:	For *i* = 1, 2, …, *n* and *b* = 1, 2, …, *B*, define $J_{bi}=I\left(\left(Y_{i},x_{i}\right)\in D_{1}^{b}\right)\in \{0,1\}$, and $J_{\cdot i}=\left(\sum_{b=1}^{B}J_{bi}\right)/B$
2:	**for** *j* = 0, 1, …, *p* **do**
3:	Compute ${\hat{V}}_{j}=\frac{n\left(n-1\right)}{{\left(n-n_{1}\right)}^{2}}\overset{n}{\underset{i=1}{\sum }}{\hat{\mathrm{cov}}}_{ij}^{2},$ where ${\hat{\mathrm{cov}}}_{ij}=\frac{1}{B}\overset{B}{\underset{b=1}{\sum }}\left(J_{bi}-J_{\cdot i}\right)\left({\tilde{\beta }}_{j}^{b}-{\hat{\beta }}_{j}\right)$
4:	Compute ${\hat{V}}_{j}^{B}={\hat{V}}_{j}-\frac{n}{B^{2}}\frac{n_{1}}{n-n_{1}}\overset{B}{\underset{b=1}{\sum }}{\left({\tilde{\beta }}_{j}^{b}-{\hat{\beta }}_{j}\right)}^{2}$
5:	**end for**
6:	Set ${\hat{V}}^{B}=\left({\hat{V}}_{1}^{B},{\hat{V}}_{2}^{B},\ldots ,{\hat{V}}_{p}^{B}\right)$

Assumption (A1) states that the predictors are uniformly bounded, which is reasonable as predictors are often normalized during data pre-processing. As defined in (A2), Σ = diag(1, Σ_*x*_), where Σ_*x*_ is the variance-covariance matrix of **x**. The boundedness of the eigenvalues of the variance-covariance matrix of **x** has been commonly assumed in the high dimensional literature (Zhao and Yu, 2006; Belloni and Chernozhukov, 2011; Fan et al., 2014; Van de Geer et al., 2014). (A3) restricts the orders of *p* and *n* as well as the sparsity of β*. Both (A1) and (A2) guarantee the convergence of the MLEs for the low dimensional GLMs (3) with a diverging number of predictors (Portnoy, 1985; He and Shao, 2000). (A3) requires S to possess the sure screening property, which relaxes the selection consistency assumption in Fei et al. (2019).

Variable selection methods that satisfy the sure screening property are available. For example, Assumptions (A1) and (A2), along with a “beta-min” condition, which stipulates that $\min_{j\in S^{*}}\left|\beta_{j}^{*}\right|>c_{0}n^{-\kappa }$ with *c*_0_ > 0, 0 < *κ* < 1/2, ensure that the commonly used sure independence screening (SIS) procedure (Fan and Song, 2010) satisfy the sure screening property; see Theorem 4 in Fan and Song (2010). While a “beta-min” condition is common in deriving the sure screening property, it is not required for the de-biased type of estimators. We take S to be the SIS procedure when conducting variable selection in simulations and the data analysis. Theorems 1 and 2 correspond to the one-time estimator and the SSGLM estimator, respectively.

**Theorem 1** *Given model (1) and assumptions (A1)—(A3), consider the one-time estimator* $\tilde{\beta }={\left({\tilde{\beta }}_{0},{\tilde{\beta }}_{1},\ldots ,{\tilde{\beta }}_{p}\right)}^{\text{T}}$ *as defined in (3). Denote p_s_* = |*S*| *and* ${\tilde{\sigma }}_{j}^{2}={\left({\left\{I_{S_{+j}}^{*}\right\}}^{-1}\right)}_{jj}$, *j* ∈ {0, 1, …, *p*}. *Then as n* → ∞,

- ${\left\|{\tilde{\beta }}_{S_{+j}}-\beta_{S_{+j}}^{*}\right\|}_{2}^{2}=o_{p}\left(p_{s}/n\right),$, *if p_s_* log *p_s_/n* → 0;
- $\sqrt{n_{1}}\left({\tilde{\beta }}_{j}-\beta_{j}^{*}\right)/{\tilde{\sigma }}_{j}\overset{d}{\to }N\left(0,1\right)$, *if* $p_{s}^{2}\log p_{s}/n\to 0$.

**Theorem 2** *Given model (1) and under assumptions (A1)—(A3) and a partial orthogonality condition that* {*x_j_, j* ∈ S*} *are independent of* {*x_k_, k* ∉ S*}, *consider the smoothed estimator* $\hat{\beta }={\left({\hat{\beta }}_{0},{\hat{\beta }}_{1},\ldots ,{\hat{\beta }}_{p}\right)}^{T}$ *as defined in (4). For each j, define* ${\overset{ˇ}{\sigma }}_{j}^{2}={\left({\left\{I_{S_{+j}^{*}}^{*}\right\}}^{-1}\right)}_{jj}$. *Then, as n, B* → ∞,

$$\sqrt{n}\left(\hat{\beta_{j}}-\beta_{j}^{*}\right)/{\overset{ˇ}{\sigma }}_{j}\overset{d}{\to }N\left(0,1\right).$$

The added partial orthogonality condition for Theorem 2 is a technical assumption for the validity of the theorem, which has been assumed in the high dimensional literature (Fan and Lv, 2008; Fan and Song, 2010; Wang and Wang, 2014). However, our numerical experiments suggest the robustness of our results to the violation of this condition. In addition, while both of the one-time estimator ${\tilde{\beta }}_{j}$ and the SSGLM estimator ${\hat{\beta }}_{j}$ possess asymptotic consistency and normality, the key advantage of ${\hat{\beta }}_{j}$ over ${\hat{\beta }}_{j}$ lies in the efficiency. An immediate observation is that ${\hat{\beta }}_{j}$ is estimated using all *n* samples but ${\tilde{\beta }}_{j}$ is estimated with only *n*_1_ samples, which explains the different normalization constants in their respective variances, ${\hat{\sigma }}_{j}^{2}/n$ and ${\tilde{\sigma }}_{j}^{2}/n_{1}$. In addition, with ${\tilde{\sigma }}_{j}^{2}$ depending solely on a one-time variable selection *S*, its variability is high given the wide variability of *S*. On the other hand, ${\hat{\sigma }}_{j}^{2}$ implicitly averages over the multiple selections, *S^b^*’s, and gains efficiency via “the effect of bagging” (Bühlmann and Yu, 2002); also see Web Table 1 of Fei et al. (2019) for empirical evidence under the linear regression setting. Moreover, the high variability of ${\tilde{\beta }}_{j}$ may lead to a large false positive rate; see Figure 1 of Fan and Lv (2008).

We defer the proofs to the Appendix, but provide some intuition here. The randomness of the selection ${\hat{S}}_{\lambda }$ presents difficulties when developing the theoretical properties, but why sure screening works is that, given any subset *S* ⊇ S*, the estimator ${\tilde{\beta }}_{S}$ is consistent, though less efficient (with additional noise variables) than the “oracle estimator” ${\tilde{\beta }}_{S^{*}}$ acting upon the true active set. The proof also shows that ${\hat{\sigma }}_{j}^{2}$ depends on the unknown S*, taking into account the variation in *B* random splits. Therefore, direct computation of ${\hat{\sigma }}_{j}^{2}$ in an analytical form is not feasible. Alternatively, we estimate the variance component via the infinitesimal jackknife method (Efron, 2014; Fei et al., 2019).

## 4. Variance Estimator and Inference by SSGLM

The infinitesimal jackknife method has been applied to estimate the variance of the bagged estimator with bootstrap-type resampling (sampling with replacement) (Efron, 2014; Fei et al., 2019). The idea is to treat each ${\tilde{\beta }}_{j}^{b}$ as a function of the sub-sample $D_{1}^{b}$, or its empirical distribution represented by the sampling indicator vector **J**_*b*_ = (*J*_*b*1_, *J*_*b*2_, …, *J_bn_*), where *J_bi_* ∈ {0, 1} is an indicator of whether the *i^th^* observation is sampled in $D_{1}^{b}$. We further denote $J_{\cdot i}=\left(\sum_{b=1}^{B}J_{bi}\right)/B$. With slightly overused notation, let

$${\tilde{\beta }}_{j}^{b}=t\left(D_{1}^{b}\right)=t\left(J_{b};D^{\left(n\right)}\right);$$

$${\hat{\beta }}_{j}=\frac{1}{B}\overset{B}{\underset{b=1}{\sum }}{\tilde{\beta }}_{j}^{b}\overset{p}{\to }E^{*}t\left(J_{b};D^{\left(n\right)}\right),\;\text{as}\;B\to \infty ,$$

where *t*(·) is a general function that maps the data to the estimator, the expectation E* and the convergence are with respect to the probability measure induced by the random ness of **J**_*b*_’s. We can generalize the infinitesimal jackknife to estimate the variance, $\text{Var}\left({\hat{\beta }}_{j}\right)$, analogous to equation (8) of Wager and Athey (2018), as follows

(5) $${\hat{V}}_{j}=\frac{n-1}{n}{\left(\frac{n}{n-n_{1}}\right)}^{2}\overset{n}{\underset{i=1}{\sum }}{\hat{\mathrm{cov}}}_{ij}^{2},$$

where

$${\hat{\mathrm{cov}}}_{ij}=\frac{1}{B}\overset{B}{\underset{b=1}{\sum }}\left(J_{bi}-J_{\cdot i}\right)\left({\tilde{\beta }}_{j}^{b}-{\hat{\beta }}_{j}\right)$$

is the covariance between the estimates ${\tilde{\beta }}_{j}^{b}’s$ and the sampling indicators *J_bi_*’s with respect to the *B* splits. Here, *n*(*n* − 1)/(*n* − *n*_1_)^2^ is a finite-sample correction term with respect to the sub-sampling scheme. Theorem 1 of Wager and Athey (2018) implies that this variance estimator is consistent, in the sense that ${\hat{V}}_{j}/\text{Var}\left({\hat{\beta }}_{j}\right)\overset{p}{\to }1$ as *B* → to ∞.

We further propose a bias-corrected version of (5):

(6) $${\hat{V}}_{j}^{B}=\hat{V_{j}}-\frac{n}{B^{2}}\frac{n_{1}}{n-n_{1}}\overset{B}{\underset{b=1}{\sum }}{\left({\tilde{\beta }}_{j}^{b}-\hat{\beta_{j}}\right)}^{2}.$$

The derivation is similar to that in Section 4.1 of Wager et al. (2014), but it is adapted to the sub-sampling scheme. The difference between ${\hat{V}}_{j}$ and ${\hat{V}}_{j}^{B}$ converges to zero with *n, B* → ∞ to, as it can be re-written as $\frac{n}{B}\frac{n_{1}}{n-n_{1}}{\hat{\upsilon }}_{j}$, where ${\hat{\upsilon }}_{j}=B^{-1}\sum_{b=1}^{B}{\left({\tilde{\beta }}_{j}^{b}-{\hat{\beta }}_{j}\right)}^{2}$ is the sample variance of ${\tilde{\beta }}_{j}^{b}’\text{s}$ from *B* splits. Thus both variance estimators are asymptotically equal. See Algorithm 2 for a summarized procedure of computing the bias-corrected variance estimates.

For finite samples, we give the order of *B* to control the Monte Carlo errors of these two variance estimators. First, with *n*_1_ = *qn* for a fixed 0 < *q* < 1, the bias of ${\hat{V}}_{j}$ is of order $n{\hat{v}}_{j}/B$ (Wager et al., 2014). Thus, setting *B* = *O*(*n*^1.5^) will reduce the bias to the desired level of *O*(*n*^−0.5^). On the other hand, ${\hat{V}}_{j}^{B}$ effectively removes this bias, as it only requires *B* = *O*(*n*) to control the Monte Carlo Mean Squared Error (MSE) to *O*(*n*^−1^) (Wager et al., 2014). A comparison between ${\hat{V}}_{j}$ and ${\hat{V}}_{j}^{B}$, given in Simulation **Example 1**, also shows the preference of ${\hat{V}}_{j}^{B}$ to ${\hat{V}}_{j}$.

For 0 < *α* < 1, the asymptotic 100(1 − *α*)% confidence interval for $\beta_{j}^{*}$, *j* = 1, …, *p*, is given by

$$\left({\hat{\beta }}_{j}-\Phi^{-1}\left(1-\alpha /2\right)\sqrt{{\hat{V}}_{j}^{B}},\;{\hat{\beta }}_{j}+\Phi^{-1}\left(1-\alpha /2\right)\sqrt{{\hat{V}}_{j}^{B}}\right),$$

and the p-value for testing $H_{0}:\beta_{j}^{*}=0$ is

$$2\times \left\{1-\Phi \left(\left|{\hat{\beta }}_{j}\right|/\sqrt{{\hat{V}}_{j}^{B}}\right)\right\},$$

where Φ is the CDF of the standard normal distribution.

## 5. Extension to Subvectors With Fixed Dimensions

We extend the SSGLM procedure to derive confidence regions for a subset of predictors and to test for contrasts of interest. Consider $\beta_{S^{\left(1\right)}}^{*}$ with |*S*^(1)^| = *p*_1_ ≥ 2, which is finite and does not increase with *n* or *p*. Accordingly, the SSGLM estimator for it is presented in Algorithm 3, and the extension of Theorem 2 is stated below.

**Theorem 3** *Given model (1) under assumptions (A1)—(A3) and a fixed finite subset S*^(1)^ ⊂ {1, 2, …, *p*} *with* |*S*(1)| = *p*_1_, let ${\hat{\beta }}^{\left(1\right)}$ *be the smoothed estimator for* $\beta_{S^{\left(1\right)}}^{*}$ *as defined in Algorithm 3. Then as n, B* → ∞,

$$\sqrt{n}I^{\left(1\right)}\left({\hat{\beta }}^{\left(1\right)}-\beta_{S^{\left(1\right)}}^{*}\right)\overset{d}{\to }N\left(0,I_{p1}\right)$$

*where* **I**_*p*1_ *is a p*_1_ × *p*_1_ *identity matrix, and I*^(1)^ *is a p*_1_ × *p*_1_ positive definite matrix depending on *S*^(1)^ and S* *and is defined in the proof*.

There is a direct extension of the one-dimensional infinitesimal jackknife for estimating the variance-covariance matrix of ${\hat{\beta }}^{\left(1\right)}$, ${\hat{\Sigma }}^{\left(1\right)}={\hat{\text{COV}}}_{\left(1\right)}^{\text{T}}{\hat{\text{COV}}}_{\left(1\right)}$, where

$${\hat{\text{COV}}}_{\left(1\right)}={\left({\hat{\text{COV}}}_{1}^{\left(1\right)},{\hat{\text{COV}}}_{2}^{\left(2\right)},\ldots ,{\hat{\text{COV}}}_{n}^{\left(1\right)}\right)}^{\text{T}},\;\text{with}\;{\hat{\text{COV}}}_{1}^{\left(1\right)}=\overset{B}{\underset{b=1}{\sum }}\left(J_{bi}-J_{i}\right)\left({\hat{\beta }}_{S^{\left(1\right)}}^{b}-{\hat{\beta }}^{\left(1\right)}\right)/B$$

To test $H_{0}:Q\beta^{\left(1\right)}=R$, where *Q* is an *r* × *p*_1_ contrast matrix and *R* is an *r* × 1 vector, a Wald-type test statistic can be formulated as

(7) $$T={\left(Q{\hat{\beta }}^{\left(1\right)}-R\right)}^{\text{T}}{\left[Q{\hat{\Sigma }}^{\left(1\right)}Q^{\text{T}}\right]}^{-1}\left(Q{\hat{\beta }}^{\left(1\right)}-R\right),$$

which follows $\chi_{r}^{2}$ under *H*_0_. Therefore, with a significance level *α* ∈ (0, 1), we reject *H*_0_ when *T* is larger than the (1 − α) × 100 percentile of $\chi_{r}^{2}$.

**Algorithm 3:** SSGLM for Subvector ***β***^(1)^

**Require:** A selection procedure $S_{\lambda }$	
**Input:** Data (**Y, X**), a data splitting proportion *q* ∈ (0, 1), the number of splits *B*, and an index set *S*^(1)^ for the predictors of interest	
**Output:** Estimates of the coefficients of predictors indexed by *S*^(1)^, ${\hat{\beta }}^{\left(1\right)}$	
1:	**for** *b* = 1, 2, …, *B* **do** Split the samples into two parts **D**_1_ and **D**_2_, with |**D**_1_| = *qn* and |**D**_2_| = (1 − *q*)*n*
2:	Apply $S_{\lambda }$ to **D**_2_ to select a subset of important predictors *S* ⊂ {1, …, *p*}
3:	Fit a GLM by regressing **Y**^1^ on $X_{S^{\left(1\right)}\cup S}^{1}$, where **D**_1_ = (**Y**^1^, **X**^1^) and compute the MLEs, denoted by ${\tilde{\beta }}^{\left(1\right)}$
4:	Define ${\tilde{\beta }}_{S^{\left(1\right)}}^{b}={\left({\tilde{\beta }}^{\left(1\right)}\right)}_{S^{\left(1\right)}}$
5:	**end for**
6:	Compute ${\hat{\beta }}^{\left(1\right)}=\left(\sum_{b=1}^{B}{\tilde{\beta }}_{S^{\left(1\right)}}^{b}\right)/B$

## 6. Simulations

We compared the finite sample performance of the proposed SSGLM procedure, under various settings, with two existing methods, the de-biased LASSO for GLMs (Van de Geer et al., 2014; Dezeure et al., 2015) and the de-correlated score test (Ning and Liu, 2017), in estimation accuracy and computation efficiency. We also investigated how the choice of *q* = *n*_1_/*n*, the splitting proportion, may impact the performance of SSGLM, explored various selection methods as part of the SSGLM procedure and their impacts on estimation and inference, illustrated our method with both logistic and Poisson regression settings, and assessed the power and type I error of the procedure. We adopted some challenging simulation settings in Bühlmann et al. (2014). For example, the indices of the active set, as well as the non-zero effect sizes, were randomly generated, and various correlation structures were explored.

**Example 1** investigated the performance of SSGLM with various splitting proportions and the convergence of the proposed variance estimators. We set *n*_1_ = *qn, q* = 0.1, 0.2, …, 0.9. Under a linear regression model, *Y_i_* = **x**_i_***β*** + *ε_i_*, *i* = 1, 2, …, *n* with i.i.d. *ε_i_* ~ *N*(0, 1), we set *n* = 500, *p* = 1,000, *s*_0_ = 10 with an AR(1) correlation structure, i.e. Σ_*ij*_ = *ρ*^|*i*−*j*|^, *ρ* = 0.5, *i, j* = 1, 2, …, *p*. The indices in the active set S* randomly varied from {1, …, *p*}, and the non-zero effects of $\beta_{j}^{*}$, *j* ∈ S* were generated from Unif[(−1.5, −0.5) ∪ (0.5, 1.5)]. For each *q*, we computed the MSE for ${\hat{\beta }}_{j}^{\left(k\right)}$, the smoothed estimate of *β_j_* from the *k*-th simulation, *k* = 1, 2, …, *K*,

$${\text{MSE}}_{j}=\frac{1}{K}\overset{K}{\underset{k=1}{\sum }}{\left({\hat{\beta }}_{j}^{\left(k\right)}-\beta_{j}^{*}\right)}^{2},\;{\text{MSE}}_{\text{avg}}=\frac{1}{p}\overset{p}{\underset{j=1}{\sum }}{\text{MSE}}_{j}.$$

**Figure 1:**
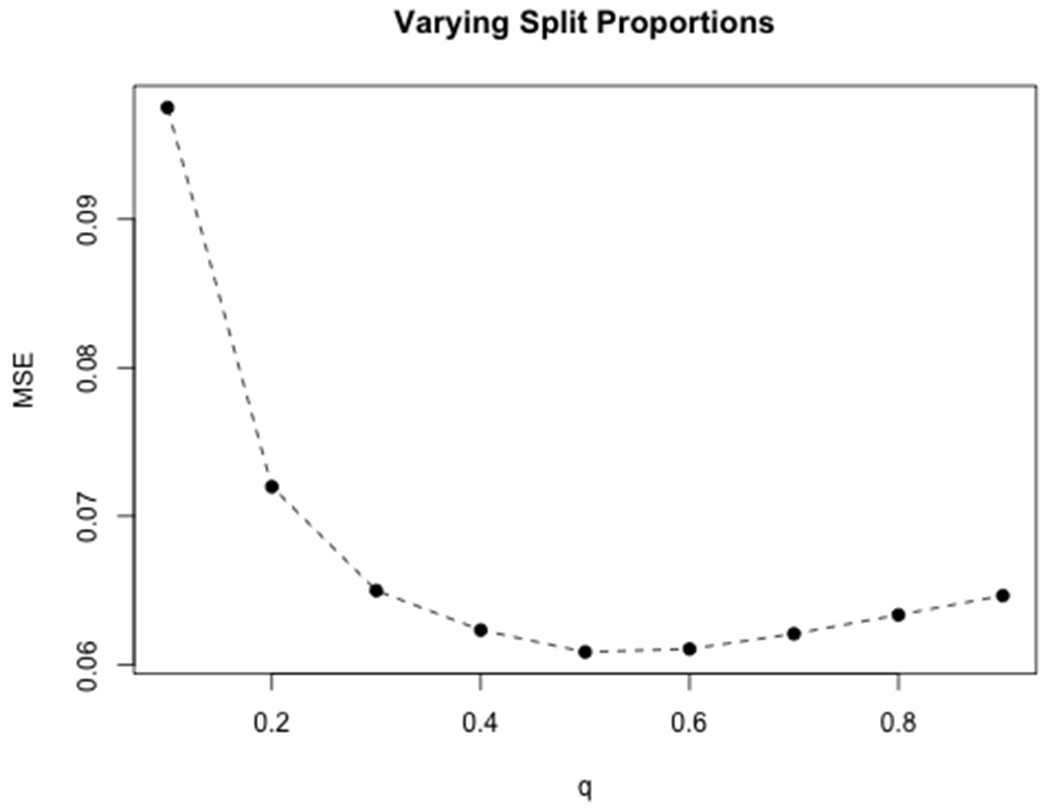

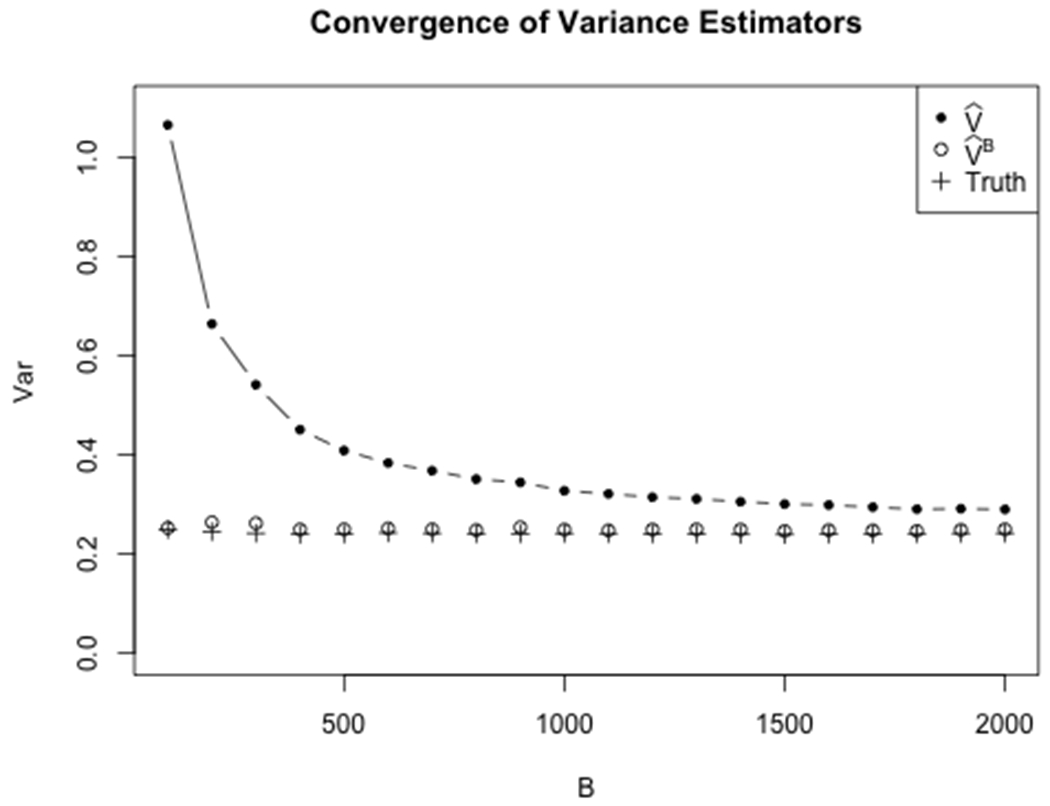
Left: Average MSEs of all predictors at split proportions *q*’s from 0.1 to 0.9. Right: Convergence of two variance estimators as *B* increases.

The left panel of Figure 1 showed that the minimum MSE was achieved when *q* = 0.5, suggesting the rationality of equal-size splitting in practice.

However, the MSE was, in general, less sensitive to *q* when *q* was getting larger, hinting that a large *n*_1_ may lead to adequate accuracy. Intuitively, there is a minimum sample size *n*_2_ = (1 − *q*)*n* required for the selections to achieve the “sure screening” property. For example, LASSO with smaller sample size would select less variables given the same tuning parameter. On the other hand, larger *n*_1_ = *qn* improves the power of the low dimensional GLM estimators directly. Thus the optimal split proportion is achieved when *n*_1_ is as large as possible, while *n*_2_ is large enough for the sure screening selection to hold. This intuition is also validated in Figure 1, as efficiency is gained faster at the beginning due to better GLM estimators with larger *n*_1_. This gain is then outweighed by the bias due to poor selections with small *n*_2_. Our conclusion is that an optimal split proportion exists, but depends on the specific selection method, the true model size, and other factors, rather than being fixed.

We further examined the convergence of the two variance estimators ${\hat{V}}_{j}$ and ${\hat{V}}_{j}^{B}$ proposed in (5) and (6) with respect to the number of splits, *B*. Under the same setting, and with *q* = 0.5, we calculated both ${\hat{V}}_{j}$ and ${\hat{V}}_{j}^{B}$ for *B* = 100, 200, …, 2,000, and compared these estimates with the empirical variance of ${\hat{\beta }}_{j}’s$ (considered to be the *truth*) based on 200 simulation replicates. The right panel of Figure 1 plots the averages over all signals *j* = 1, 2, …, *p* and shows ${\hat{V}}_{j}$ converges to the truth much slower than ${\hat{V}}_{j}^{B}$, and ${\hat{V}}_{j}^{B}$ has small biases even with a relatively small *B*.

**Example 2** implemented various selection methods, LASSO, SCAD, MCP, Elastic net, and Bayesian LASSO, when conducting variable selection for SSGLM, and compared their impacts on estimation and inference. Ten-fold cross-validation was used for the tuning parameters in each selection procedure. We assumed a Poisson model with *n* = 300, *p* = 400, and *s*_0_ = 5. For *i* = 1, …, *n*,

(8) $$\log\left(E\left(Y_{i}|x_{i}\right)\right)=\beta_{0}+x_{i}\beta .$$

Table 1 reports the selection frequency for each *j* out of *B* splits. Larger $\left|\beta_{j}^{*}\right|$ yielded a higher selection frequency. For example, predictors with an absolute effect larger than 0.6 were selected frequently. The average size of the selected models by each method varied from 23 (for LASSO) to 8 (for Bayesian LASSO). However, in terms of the bias, coverage probabilities, and mean squared errors, the impact of the different variable selection methods seemed to be negligible. Thus, SSGLM was fairly robust to the choice of variable selection method.

**Example 3** also assumed model (8). We set *n* = 400, *p* = 500, and *s*_0_ = 6, with non-zero coefficients between 0.5 and 1, and three correlation structures: Identity; AR(1) with $\Sigma_{ij}=\rho^{\left|i-j\right|}$, *ρ* = 0.5; Compound Symmetry (CS) with $\Sigma_{ij}=\rho^{I\left(i\neq j\right)}$, *ρ* = 0.5.

Table 2 shows that SSGLM consistently provided nearly unbiased estimates. The obtained standard errors (SEs) were close to the empirical standard deviations (SDs), leading to confidence intervals with coverage probabilities that were close to the 95% nominal level.

**Example 4** assumed a logistic regression model for binary outcomes, with *n* = 400, *p* = 500, and *s*_0_ = 4,

(9) $$\text{logit}\left(P\left(Y_{i}=1|x_{i}\right)\right)=\beta_{0}+x_{i}\beta .$$

The index set for predictors with nonzero coefficients, S* = {218, 242, 269, 417}, were randomly generated, and $\beta_{S^{*}}^{*}=(-2,-1,1,2)$. We report the performance of SSGLM when inferring the subvector $\beta_{S^{*}}^{*}$, in Tables 3 and 4. Our method gave nearly unbiased estimates under different correlation structures and sufficient power for the various contrasts.

**Example 5** compared our method with the de-biased LASSO estimator (Van de Geer et al., 2014) and the de-correlated score test (Ning and Liu, 2017) in terms of power and type I error. We assumed model (9) with *n* = 200, *p* = 300, *s*_0_ = 3, and $\beta_{S^{*}}^{*}=(2,-2,2)$ with AR(1) correlation structures. Table 5 summarises the power of detecting each true signal and the average type I error for the noise variables under the AR(1) correlation structure with four correlation values, *ρ* = 0.25, 0.4, 0.6, 0.75.

Our method was shown to be the most powerful, while maintaining the type I error around the nominal 0.05 level. The power was over 0.9 for the first three scenarios and was above 0.8 with *ρ* = 0.75. The de-biased LASSO estimators controlled the type I error well, but the power dropped from 0.9 to approximately 0.67 as the correlation among the predictors increased. The de-correlated score tests had the least power and the highest type I error. While these two competing methods have the same efficiency asymptotically, they do differ by specific implementations, for example, the choice of tuning parameters. Indeed, de-biased methods may be sensitive to tuning parameters, which could explain the gap in the finite sample performance.

Table 5 summarizes the average computing time (in seconds) of the three methods per data set (R-3.6.2 on an 8-core MacBook Pro). On average, our method took 17.7 seconds, which was the fastest among the three methods. The other two methods were slower for the smaller *ρ*’s (75 and 37 seconds, respectively) and faster for the larger *ρ*’s (41 and 18 seconds, respectively), likely because the node-wise LASSO procedure that was used for estimating the precision matrix tended to be faster when handling more highly correlated predictors.

## 7. Data Example

We analyzed a subset of the BLCSC data (Christiani, 2017), consisting of *n* = 1,459 individuals, among whom 708 were lung cancer patients and 751 were controls. After cleaning, the data contained 6,829 SNPs, along with important demographic variables including age, gender, race, education level, and smoking status (Table 6). As smoking is known to play a significant role in the development of lung cancer, we were particularly interested in estimating the interactions between the SNPs and smoking status, in addition to their main effects.

We assumed a high-dimensional logistic model with the binary outcome being an indicator of lung cancer status. Predictors included demographic variables, the SNPs (with prefix “AX”), and the interactions between the SNPs and smoking status (with prefix “SAX”; *p* = 13,663). We applied the SSGLM with *B* = 1, 000 random splits and drew inference on all 13,663 predictors. Table 7 lists the top predictors ranked by their p-values. We identified 9 significant coefficients after using Bonferroni correction for multiple comparisons. All were interaction terms, providing strong evidence of SNP-smoking interactions, which have rarely been reported. These nine SNPs came from three genes, TUBB, ERBB2, and TYMS. TUBB mutations are associated with both poor treatment response to paclitaxel-containing chemotherapy and poor survival in patients with advanced non-small-cell lung cancer (NSCLC) (Monzo et al., 1999; Kelley et al., 2001). Rosell et al. (2001) has proposed using the presence of TUBB mutations as a basis for selecting initial chemotherapy for patients with advanced NSCLC. In contrast, intragenic ERBB2 kinase mutations occur more often in the adenocarcinoma lung cancer subtype (Stephens et al., 2004; Beer et al., 2002). Lastly, advanced NSCLC patients with low/negative thymidylate synthase (TYMS) are shown to have better responses to Pemetrexed–based chemotherapy and longer progression free survival (Wang et al., 2013).

For comparisons, we applied the de-sparsified estimator for GLM (Bühlmann et al., 2014). A direct application of the “lasso.proj” function in the “hdi” R package (Dezeure et al., 2015) was not feasible given the data size. Instead, we used a shorter sequence of candidate *λ* values and 5-fold instead of 10-fold cross validation for the node-wise LASSO procedure. This procedure costs approximately one day of CPU time. After correcting for multiple testing, there were two significant coefficients, both of which were interaction terms corresponding to SNPs AX.35719413_C and AX.83477746_A. Both SNPs were from the TUBB gene, and the first SNP was also identified by our method.

**Figure 2:**
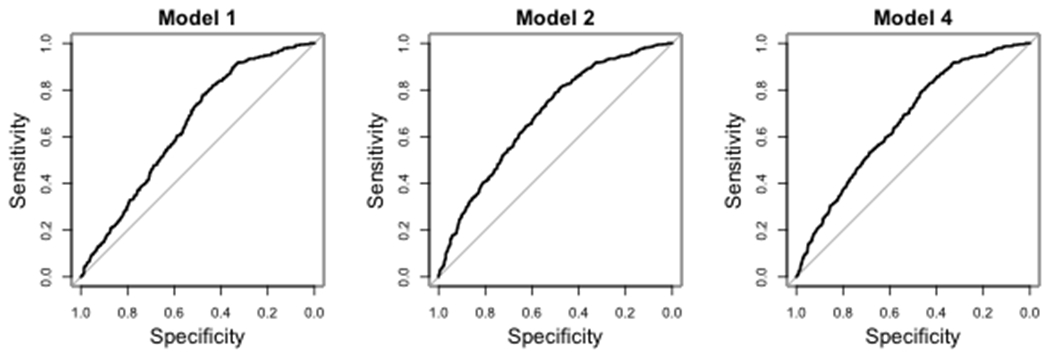
ROC curves of the three selected models.

To validate our findings, we applied the prediction accuracy measures for nonlinear models proposed in Li and Wang (2019). We calculated the *R*^2^, the proportion of variation explained in **Y**, for the models we chose to compare. We report five models and their corresponding *R*^2^ values: **Model 1**. the baseline model including only the demographic variables (*R*^2^ = 0.0938); **Model 2**. the baseline model plus the significant interactions after the Bonferroni correction in Table 7 (*R*^2^ = 0.1168); **Model 3**. Model 2 plus the main effects of its interaction terms (*R*^2^ = 0.1181); **Model 4**. the baseline model plus the significant interactions from the de-sparsified LASSO method (*R*^2^ = 0.1018); **Model 5**. Model 4 plus the corresponding main effects (*R*^2^ = 0.1076). Model 2 based on our method explained 25% more variation in **Y** than the baseline model (from 0.0938 to 0.1168), while Model 4 based on the de-sparsified LASSO method only explains 8.5% more variation (from 0.0938 to 0.1018). We also plotted Receiver-Operating Characteristic (ROC) curves for models 1, 2, and 4 (Figure 2). Their corresponding areas under the curves (AUCs) were 0.645, 0.69, and 0.668, respectively.

Previous literature has identified several SNPs as potential risk factors for lung cancer. We studied a controversial SNP, rs3117582, from the TUBB gene on chromosome 6. This SNP was identified in association with lung cancer risk in a case/control study by Wang et al. (2008), while on the other hand, Wang et al. (2009) found no evidence of association between the SNP and risk of lung cancer among *never-smokers*. Our goal was to test this SNP and its interaction with smoking in the presence of all the other predictors under the high dimensional logistic model. Slightly overusing notation, we denoted the coefficients corresponding to rs3117582 and its interaction with smoking as ***β***^(1)^ = (*β*_1_, *β*_2_), and tested *H*_0_ : *β*_1_ = *β*_2_ = 0. Applying the proposed method, we obtained

$$\left({\hat{\beta }}_{1},{\hat{\beta }}_{2}\right)=\left(-0.067,0.005\right),\hat{\mathrm{COV}}\left({\hat{\beta }}_{1},{\hat{\beta }}_{2}\right)=\left(\begin{array}{cc} 0.44 & -0.43\\ -0.43 & 0.50 \end{array}\right)$$

The test statistic of the overall effect was *T* = 0.062 by (7) with a p-value of 0.97, which concluded that, among the patients in BLCSC, rs3117582 was not significantly related to lung cancer, regardless of the smoking status.

## 8. Conclusions

Our approach for drawing inference, by adopting a “split and smoothing” idea, improves upon Fei et al. (2019) which used bootstrap resampling, and recasts a high dimensional inference problem into a sequence of low dimensional estimations. Unlike many of the existing methods (Zhang and Zhang, 2014; Bühlmann et al., 2014; Javanmard and Montanari, 2018), our method is more computationally feasible as it does not require estimating high dimensional precision matrices. Our algorithm enables us to make full use of parallel computing for improved computational efficiency, because fitting the *p* low dimensional GLMs and randomly splitting the data *B* times are both separable tasks, which can be implemented in parallel.

We have derived the variance estimator using the infinitesimal jackknife method adapted to the splitting and smoothing procedure (Efron, 2014; Wager and Athey, 2018). This estimator is free of parametric assumptions, resembles bagging (Bühlmann and Yu, 2002), and leads to confidence intervals with correct coverage probabilities. Moreover, we have relaxed the stringent *selection consistency* assumption on variable selection as required in Fei et al. (2019). We have shown that our procedure works with a mild *sure screening* assumption for the selection method.

There are open problems to be addressed. First, our method relies on a sparsity condition for the model parameters. We envision that relaxation of the condition may take a major effort, though our preliminary simulations (Example B.2 in Appendix B) suggest that our procedure might work when the sparsity condition fails. Second, as our model is fully parametric, in-depth research is needed to develop a more robust approach when the model is mis-specified. Finally, while our procedure is feasible when *p* is large (tens of thousands), the computational cost increases substantially when *p* is extraordinarily large (millions). Much effort is warranted to enhance its computational efficiency. Nevertheless, our work does provide a starting point for future investigations.

## Figures and Tables

**Table 1:** Comparisons of different selection procedures to implement our proposed method. The first column is the indices of the non-zero signals. The last row for the selection frequency is the average number of predictors being selected by each procedure. The last row for the coverage probability is the average coverage probability of all predictors.

Index *j*	$\beta_{j}^{*}$	LASSO	SCAD	MCP	EN	Bayesian
Selection frequency						
12	0.4	0.59	0.55	0.49	0.60	0.60
71	0.6	0.93	0.92	0.90	0.95	0.94
351	0.8	0.99	0.99	0.99	1.00	1.00
377	1.0	1.00	1.00	1.00	1.00	1.00
386	1.2	1.00	1.00	1.00	1.00	1.00
Average model size		23.12	13.15	10.89	10.31	7.98

Bias						
12	0.4	0.003	0.003	0.003	0.003	0.001
71	0.6	0.007	0.008	0.008	0.008	−0.010
351	0.8	−0.001	0.001	0	0	0.001
377	1.0	−0.005	−0.005	−0.006	−0.005	0.001
386	1.2	0.002	0.001	0.001	0.001	0.004

Coverage probability						
12		0.90	0.90	0.91	0.91	0.95
71		0.94	0.94	0.95	0.94	0.94
351		0.95	0.95	0.95	0.94	0.95
377		0.94	0.93	0.93	0.94	0.92
386		0.94	0.95	0.95	0.95	0.94
Average		0.93	0.94	0.94	0.94	0.94

MSE						
12		0.111	0.110	0.110	0.109	0.106
71		0.104	0.103	0.102	0.102	0.101
351		0.103	0.103	0.103	0.103	0.100
377		0.101	0.100	0.100	0.100	0.109
386		0.097	0.096	0.096	0.096	0.102
Average		0.105	0.104	0.103	0.103	0.102

**Table 2:** SSGLM under the Poisson regression and three correlation structures. Bias, average standard error (SE), empirical standard deviation (SD), coverage probability (Cov prob), and selection frequency (Sel freq) are reported. The last column summarizes the average of all noise variables.

	Index *j*	0 (Int)	74	109	347	358	379	438	-
	$\beta_{j}^{*}$	1.000	0.810	0.595	0.545	0.560	0.665	0.985	0

Identity	Bias	−0.010	0	0	0.001	0.005	0.005	0.006	0
	SE	0.050	0.035	0.034	0.035	0.035	0.034	0.035	0.034
	SD	0.064	0.036	0.038	0.031	0.033	0.038	0.036	0.036
	Cov prob	0.870	0.920	0.900	0.960	0.990	0.910	0.950	0.936
	Sel freq	1.000	1.000	1.000	1.000	1.000	1.000	1.000	0.015

AR(1)	Bias	0.006	0.003	−0.002	−0.001	−0.001	−0.005	0.003	0
	SE	0.052	0.035	0.035	0.035	0.035	0.035	0.035	0.035
	SD	0.056	0.031	0.041	0.035	0.037	0.037	0.037	0.036
	Cov prob	0.930	0.970	0.890	0.960	0.950	0.930	0.960	0.937
	Sel freq	1.000	1.000	1.000	1.000	1.000	1.000	1.000	0.015

CS	Bias	−0.003	−0.005	0.004	−0.002	0.005	−0.004	−0.001	0.001
	SE	0.033	0.043	0.043	0.042	0.043	0.043	0.044	0.042
	SD	0.038	0.046	0.044	0.052	0.040	0.047	0.043	0.044
	Cov prob	0.960	0.900	0.930	0.900	0.970	0.910	0.950	0.934
	Sel freq	1.000	1.000	0.999	0.997	0.998	0.999	1.000	0.016

**Table 3:** SSGLM under the logistic regression, with estimation and inference for the subvector ***β***^(1)^ = ***β***_S*_. We compare the SSGLM (left) to the oracle model (right), where the oracle estimator is from the low dimensional GLM given the true set S*, and the empirical covariance matrix is with respect to the simulation replications.

Index *j*	218	242	269	417	Index *j*	218	242	269	417
$\beta_{j}^{*}$	−2	−1	1	2	$\beta_{j}^{*}$	−2	−1	1	2

Identity									
${\hat{\beta }}^{\left(1\right)}$	−2.048	−1.043	0.999	2.096	Oracle	−1.995	−1.026	0.973	2.043
${\hat{\Sigma }}^{\left(1\right)}$	0.146	0.010	−0.009	−0.020	Empirical	0.155	0.006	−0.009	−0.027
	0.010	0.134	−0.004	−0.011		0.006	0.129	−0.011	−0.015
	−0.009	−0.004	0.134	0.009		−0.009	−0.011	0.152	0.010
	−0.020	−0.011	0.009	0.143		−0.027	−0.015	0.010	0.134

AR(1)									
${\hat{\beta }}^{\left(1\right)}$	−2.073	−1.014	1.002	2.110	Oracle	−2.024	−0.991	0.977	2.062
${\hat{\Sigma }}^{\left(1\right)}$	0.145	0.012	−0.011	−0.023	Empirical	0.141	0.012	−0.016	−0.028
	0.012	0.137	−0.006	−0.011		0.012	0.112	−0.006	0
	−0.011	−0.006	0.135	0.010		−0.016	−0.006	0.129	0.009
	−0.023	−0.011	0.010	0.147		−0.028	0	0.009	0.136

CS									
${\hat{\beta }}^{\left(1\right)}$	−2.095	−1.033	1.070	2.102	Oracle	−2.037	−1.024	1.027	2.028
${\hat{\Sigma }}^{\left(1\right)}$	0.223	−0.026	−0.048	−0.063	Empirical	0.192	−0.030	−0.044	−0.045
	−0.026	0.208	−0.043	−0.047		−0.030	0.187	−0.037	−0.044
	−0.048	−0.043	0.207	−0.028		−0.044	−0.037	0.165	−0.011
	−0.063	−0.047	−0.028	0.224		−0.045	−0.044	−0.011	0.179

**Table 4:** SSGLM under Logistic regression, with rejection rates of testing the contrasts. When the truth is 0, the rejection rates estimate the type I error probability; when the truth is nonzero, they estimating the testing power.

*H*_0_	Truth	Identity	AR(1)	CS
$\beta_{218}^{*}+\beta_{417}^{*}=0$	0	0.05	0.04	0.03
$\beta_{242}^{*}+\beta_{269}^{*}=0$	0	0.06	0.04	0.025
$\beta_{218}^{*}+\beta_{269}^{*}=0$	−1	0.56	0.57	0.42
$\beta_{242}^{*}+\beta_{417}^{*}=0$	1	0.55	0.58	0.48
$\beta_{242}^{*}=0$	−1	0.83	0.80	0.61
$\beta_{269}^{*}=0$	1	0.74	0.81	0.70
$\beta_{218}^{*}=0$	−2	1	1	1
$\beta_{417}^{*}=0$	2	1	1	1

**Table 5:** Comparisons of SSGLM, Lasso-pro, and De-correlated score test (Dscore) in power, type I error and computing time. AR(1) correlation structure with different *ρ*’s for **X** are assumed.

	Power			Type I error	Time

Truth	$\beta_{10}^{*}=2$	$\beta_{20}^{*}=-2$	$\beta_{30}^{*}=2$	$\beta_{j}^{*}=2$	(secs)
*ρ* = 0.25 Proposed	0.920	0.930	0.950	0.049	17.7
Lasso-pro	0.900	0.930	0.900	0.042	74.7
Dscore	0.790	0.880	0.890	0.177	37.0

*ρ* = 0.4 Proposed	0.940	0.960	0.965	0.049	17.6
Lasso-pro	0.920	0.910	0.920	0.043	66.0
Dscore	0.770	0.905	0.840	0.175	30.7

*ρ* = 0.6 Proposed	0.940	0.950	0.880	0.054	17.7
Lasso-pro	0.850	0.750	0.850	0.045	53.3
Dscore	0.711	0.881	0.647	0.268	20.1

*ρ* = 0.75 Proposed	0.863	0.847	0.923	0.060	17.7
Lasso-pro	0.690	0.670	0.650	0.053	41.0
Dscore	0.438	0.843	0.530	0.400	17.9

**Table 6:** Demographic characteristics of the BLCSC SNP data.

	Controls (751)	Cases (708)
Race		
White	726	668
Black	5	22
Other	20	18

Education		
<High school	64	97
High school	211	181
>High school	476	430

Age		
Mean(sd)	59.7(10.6)	60(10.8)

Gender		
Female	460	437
Male	291	271

Pack years		
Mean(sd)	18.8(25.1)	46.1(38.4)

Smoking		
Ever	498	643
Never	253	65

**Table 7:** SSGLM fitted to the BLCSC data. SNP variables start with “AX”; interaction terms start with “SAX”; “Smoke” is binary (1=ever smoked, 0=never smoked). Rows are sorted by p-values.

Variable	$\hat{\beta }$	SE	T	p-value	Adjusted P	Sel freq
SAX.88887606_T	0.33	0.02	17.47	< 10^−3^	< 0.01	0.08
SAX.11279606-T	0.53	0.06	8.23	< 10^−3^	< 0.01	0.00
SAX.88887607_T	0.29	0.04	6.97	< 10^−3^	< 0.01	0.01
SAX.15352688_C	0.56	0.08	6.90	< 10^−3^	< 0.01	0.01
SAX.88900908_T	0.54	0.09	5.95	< 10^−3^	< 0.01	0.02
SAX.88900909_T	0.51	0.09	5.69	< 10^−3^	< 0.01	0.02
SAX.32543135_C	0.78	0.14	5.49	< 10^−3^	< 0.01	0.25
SAX.11422900_A	0.32	0.06	5.24	< 10^−3^	< 0.01	0.09
SAX.35719413_C	0.47	0.10	4.63	< 10^−3^	0.049	0.00

SAX.88894133_C	0.43	0.10	4.53	< 10^−3^	0.08	0.00
SAX.11321564_T	0.47	0.11	4.44	< 10^−3^	0.12	0.00
…						
AX.88900908_T	0.40	0.11	3.84	< 10^−3^	1.00	0.00
Smoke	0.89	0.23	3.82	< 10^−3^	1.00	-
…						

**Figure 3:**
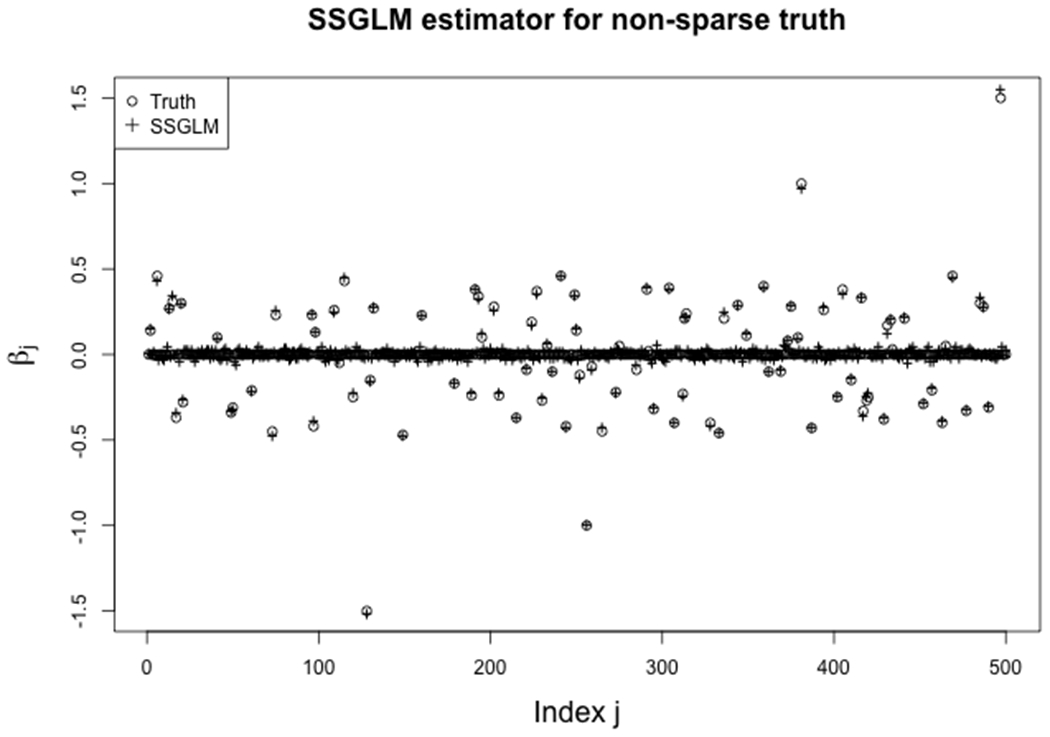
SSGLM under non-sparse truth, with *p* = 500 and *s*_0_ = 100.

## Appendix A: Proofs of Theorems

**Proof** of Theorem 1:

From the data split, **D**_1_ and **D**_2_ are mutually exclusive, thus *S*, from **D**_2_, is independent of **D**_1_ = (**Y**^1^, **X**^1^). We show the asymptotics of ${\tilde{\beta }}_{S_{+j}}$ in (3) with a diverging number of parameters *p_s_*, by using the techniques and results from He and Shao (2000); Niemiro (1992). Without loss of generality, and to simplify notation, we let *j* = 1 ∈ *S*. Then *S*_+*j*_ = *S*. The argument is the same if *1* ∉ *S* and for any other *j*.

To proceed, we first restrict on the event of Ω = {*S* ⊇ *S**}. With Assumption (A3), P(Ω) ≥ 1 − *K*_2_(*p* ∨ *n*_2_)^−1−*c*_2_^. Recall that

$${\tilde{\beta }}_{S_{+j}}=\underset{\beta \in R^{\left|S\right|+1}}{\mathrm{argmin}}\;\ell_{S}\left(\beta_{S}\right)=\underset{\beta \in R^{\left|S\right|+1}}{\mathrm{argmin}}\;\ell \left(\beta_{S};Y^{1},X_{S}^{1}\right);\;{\tilde{\beta }}_{1}={\left({\tilde{\beta }}_{S_{+j}}\right)}_{1}.$$

To apply Theorems 2.1 and 2.2 of He and Shao (2000) in the GLM case, we can verify that our Assumptions (A1) and (A2) will lead to their conditions (C1), (C2), (C4) and (C5) with *C* = 1, *r* = 2 and *A*(*n, p_s_*) = *p_s_*. To verify their (C3), we first note that their *D_n_* is our $I_{S}^{*}$. Then for any ***β**_S_*, *α* ∈ **R**^*p_s_*^ such that ‖*α*‖_2_ = 1, a second order Taylor expansion of *U_S_*(***β**_S_*) around $\beta_{S}^{*}$ leads to

$$\left|\alpha^{T}E_{\beta^{*}}\left(U_{S}\left(\beta_{S}\right)-U_{S}\left(\beta_{S}^{*}\right)\right)-\alpha^{\text{T}}I_{S}^{*}\left(\beta_{S}-\beta_{S}^{*}\right)\right|\leq O\left({\left\|\beta_{S}-\beta_{S}^{*}\right\|}_{2}^{2}\right).$$

Hence,

$$\underset{\left\|\beta_{S}-\beta_{S}^{*}\right\|\leq {\left(p_{s}/n\right)}^{1/2}}{\sup}\left|\alpha^{\text{T}}E_{\beta^{*}}\left(U_{S}\left(\beta_{S}\right)-U_{S}\left(\beta_{S}^{*}\right)\right)-\alpha^{\text{T}}I_{S}^{*}\left(\beta_{S}-\beta_{S}^{*}\right)\right|\leq O\left(p_{s}/n\right)=o\left(n^{1/2}\right),$$

which means their (C3) follows. Thus, by Theorem 2.1 of He and Shao (2000),

$${\left\|{\tilde{\beta }}_{S}-\beta_{S}^{*}\right\|}_{2}^{2}=o_{p}\left(p_{s}/n_{1}\right),$$

given *p_s_* log *p_s_/n*_1_ → 0. Furthermore, by Theorem 2.2 of He and Shao (2000), if $p_{s}^{2}\text{log}p_{s}/n_{1}\to 0$, then

$${\left\|n_{1}^{1/2}\left({\tilde{\beta }}_{S}-\beta_{S}^{*}\right)+n_{1}^{-1/2}{\left\{I_{S}^{*}\right\}}^{-1}U_{S}\left(\beta_{S}^{*}\right)\right\|}_{2}=o_{p}\left(1\right).$$

Releasing the Restriction on Ω and with P(Ω^*c*^) = P(*S* ⊇ *S**) ≤ *K*_2_(*p* ∨ *n*_2_)^−1−*c*_2_^, we would still have ${\left\|{\tilde{\beta }}_{S}-\beta_{S}^{*}\right\|}_{2}^{2}=o_{p}\left(p_{s}/n_{1}\right)$, given *p_s_* log *p_s_*/*n*_1_ → 0 . To see this, for any *ϵ* > 0, we can consider

$$\text{P}\left({\left\|{\left(n_{1}/p_{s}\right)}^{1/2}\left({\tilde{\beta }}_{S}-\beta_{S}^{*}\right)\right\|}_{2}>\epsilon \right)\;<\text{P}\left({\left\|{\left(n_{1}/p_{s}\right)}^{1/2}\left({\tilde{\beta }}_{S}-\beta_{S}^{*}\right)\right\|}_{2}>\epsilon |\Omega \right)\text{P}\left(\Omega \right)+\text{P}\left(\Omega^{c}\right)\;<\text{P}\left({\left\|{\left(n_{1}/p_{s}\right)}^{1/2}\left({\tilde{\beta }}_{S}-\beta_{S}^{*}\right)\right\|}_{2}>\epsilon |\Omega \right)+K_{2}{\left(p\vee n_{2}\right)}^{-1-c_{2}},$$

where both terms in the last inequality converge to 0 as *n*_1_ → ∞ to and *n*_2_ = (1 − *q*)*n*_1_/*q*, with 0 < *q* < 1 a constant. Similarly, we can show ${\left\|n_{1}^{1/2}\left({\tilde{\beta }}_{S}-\beta_{S}^{*}\right)+n_{1}^{-1/2}{\left\{I_{S}^{*}\right\}}^{-1}U_{S}\left(\beta_{S}^{*}\right)\right\|}_{2}=o_{p}\left(1\right)$, if $p_{s}^{2}\log p_{s}/n_{1}\to 0$, which can also be written as

(10) $${\tilde{\beta }}_{S}-\beta_{S}^{*}=-n_{1}^{-1}{\left\{I_{S}^{*}\right\}}^{-1}U_{S}\left(\beta_{S}^{*}\right)+r_{n_{1}},$$

with ${\left\|r_{n_{1}}\right\|}_{2}^{2}=o_{p}\left(1/n_{1}\right)$. Consequently, by taking *α* = (0, 1, 0, …, 0)^T^ and left-multiplying both sides of (10) by *n*^1/2^ *α*^T^, we have

$$\sqrt{n_{1}}\left({\tilde{\beta }}_{1}-\beta_{1}^{*}\right)/{\tilde{\sigma }}_{1}\overset{d}{\to }N\left(0,1\right)$$

where ${\tilde{\sigma }}_{1}^{2}={\left({\left\{I_{S}^{*}\right\}}^{-1}\right)}_{11}$. ■

The following lemma, which is needed for the proof of Theorem 2, bounds the estimates of coefficients, when the selected subset *S^b^* misses important predictors in *S** for some 1 ≤ *b* ≤ *B*. Although *S** ⊈ *S^b^* with probability going to zero by Assumption (A3), we need to establish an upper bound in order to control the bias of ${\hat{\beta }}_{j}$ for any *j*.

**Lemma 4** *With model (1) and Assumptions (A1) and (A2), consider the GLM estimator* ${\tilde{\beta }}_{S}$ *with respect to subset S as defined in (3). Denote by p_s_* = |*S*|. *If p_s_* log *p_s_*/*n* → 0, *then with probability going to 1*, ${\left|{\tilde{\beta }}_{S}\right|}_{\infty }\leq C_{\beta }$, *where C_β_* > 0 *is a constant depending on c*_min_, *c*_max_, *c_β_*, and *A*(0).

**Proof** By definition,

$${\tilde{\beta }}_{S_{+j}}=\underset{\beta_{S}\in R^{p_{s}+1}}{\mathrm{argmin}}\ell_{S}\left(\beta_{S}\right)=\underset{\beta_{S}\in R^{p_{s}+1}}{\mathrm{argmin}}\ell \left(\beta_{S};Y^{1}{,X}_{S}^{1}\right).$$

If *S** ⊆ *S*, the result immediately follows from Theorem 1 by taking *C_β_* = 2*c_β_*. When *S** ⊈ *S*, the minimizer ${\tilde{\beta }}_{S_{+j}}$ is not an unbiased estimator of $\beta_{S}^{*}$ anymore. However, we show that the boundedness of ${\tilde{\beta }}_{S_{+j}}$ is guaranteed from the strong convexity of the objective function *ℓ_S_*(***β***_S_) .

To see this, we note that the observed information is ∇2ℓS(βS)=I^S(βS)=1nX_SVTSX_S, where $V_{S}=\text{diag}\left\{A^{\prime \prime }\left({\bar{x}}_{1S}\beta_{S}\right),\ldots ,A^{\prime \prime }\left({\bar{x}}_{nS}\beta_{S}\right)\right\}$ consisting of all positive diagonal entries, because of the positivity assumption on *A*″(·). Then for any column vector *w* ∈ **R**^*p_s_*+1^, $V_{S}^{1/2}{\bar{X}}_{S}w=0$ if and only if ${\bar{X}}_{S}w=0$, implying that the positive definiteness of ∇^2^*ℓ_S_*(***β**_S_*) is equivalent to that of Σ^S=1nX_SX_TS. On the other hand, with *p_s_* log *p_s_*/*n* → 0, Lemma 1 of Fei et al. (2019) implies that, with probability going to 1, $\left\|{\hat{\Sigma }}_{S}-\Sigma_{S}\right\|\leq \varepsilon$ for *ε* = min(1/2, *c*_min_/2), and, hence,

$$\lambda_{\min}\left({\hat{\Sigma }}_{S}\right)\geq \lambda_{\min}\left(\Sigma_{S}\right)-\varepsilon \geq \lambda_{\min}\left(\Sigma \right)-\varepsilon \geq c_{\min}/2>0.$$

Thus, with probability going to 1, ${\hat{\Sigma }}_{S}$ is positive definite, yielding that

$$\ell_{S}\left(\beta_{S}\right)=n^{-1}\overset{n}{\underset{i=1}{\sum }}\left\{A\left({\bar{x}}_{iS}\beta_{S}\right)-Y_{i}{\bar{x}}_{iS}\beta_{S}\right\}$$

is strongly convex with respect to ***β**_S_*. Hence, ${\tilde{\beta }}_{S_{+j}}\in \left\{\beta_{S}:\ell_{S}\left(\beta_{S}\right)\leq A\left(0\right)\right\}$, which is a strongly convex set with probability going to 1. As *A*(0) does not depend on *S* or the data, there exists a constant *C_β_* > 0 (which only depends on *A*(0), but does not depend on *S* or the data), such that ${\left|{\tilde{\beta }}_{S}\right|}_{\infty }\leq C_{\beta }$ holds with probability going to 1. ■

**Proof** of Theorem 2:

We define the *oracle* estimators of $\beta_{j}^{*}$ on the full data (**Y**, **X**) and the *b*-th subsample $D_{1}^{b}$ respectively, where the candidate set is the true set *S**:

$${\overset{ˇ}{\beta }}_{S_{+j}^{*}}=\underset{\beta \in R^{s_{0}+1}}{\mathrm{argmin}}\ell_{S_{+j}^{*}}\left(\beta_{S_{+j}^{*}}\right)=\underset{\beta \in R^{s_{0}+1}}{\mathrm{argmin}}\ell_{S_{+j}^{*}}\left(\beta_{S_{+j}^{*}};Y,X_{S_{+j}^{*}}\right),{\overset{ˇ}{\beta }}_{j}={\left({\overset{ˇ}{\beta }}_{S_{+j}^{*}}\right)}_{j};$$

$${\overset{ˇ}{\beta }}_{S_{+j}^{*}}^{b}=\underset{\beta \in R^{s_{0}+1}}{\mathrm{argmin}}\ell_{S_{+j}^{*}}^{b}\left(\beta_{S_{+j}^{*}}\right)=\underset{\beta \in R^{s_{0}+1}}{\mathrm{argmin}}\ell_{S_{+j}^{*}}\left(\beta_{S_{+j}^{*}};Y^{1\left(b\right)},X_{S_{+j}^{*}}^{1\left(b\right)}\right),{\overset{ˇ}{\beta }}_{j}^{b}={\left({\overset{ˇ}{\beta }}_{S_{+j}^{*}}^{b}\right)}_{j}.$$

By Theorem 1 and given $s_{0}^{2}\log s_{0}/n\to 0$, for each *j* ∈ {1, …, *p*},

(11) $$\sqrt{n}\left({\overset{ˇ}{\beta }}_{j}-\beta_{j}^{*}\right)/{\overset{ˇ}{\sigma }}_{j}\overset{d}{\to }N\left(0,1\right)\;\text{as}\;n\to \infty ,$$

where ${\overset{ˇ}{\sigma }}_{j}^{2}={\left({\left\{I_{S_{+j}^{*}}^{*}\right\}}^{-1}\right)}_{jj}$.

With the oracle estimators ${\overset{ˇ}{\beta }}_{j}’\text{s}$’s and ${\overset{ˇ}{\beta }}_{j}^{b}’\text{s}$, we have the following decomposition:

(12) $$\sqrt{n}\left({\hat{\beta }}_{j}-\beta_{j}^{*}\right)\;=\sqrt{n}\left({\overset{ˇ}{\beta }}_{j}-\beta_{j}^{*}\right)+\sqrt{n}\left({\hat{\beta }}_{j}-{\overset{ˇ}{\beta }}_{j}\right)\;=\sqrt{n}\left({\overset{ˇ}{\beta }}_{j}-\beta_{j}^{*}\right)+\sqrt{n}\left(\frac{1}{B}\overset{B}{\underset{b=1}{\sum }}{\tilde{\beta }}_{j}^{b}-{\overset{ˇ}{\beta }}_{j}\right)\;=\underset{\text{I}}{\underset{︸}{\sqrt{n}\left({\overset{ˇ}{\beta }}_{j}-\beta_{j}^{*}\right)}}+\underset{\text{II}}{\underset{︸}{\sqrt{n}\left(\frac{1}{B}\overset{B}{\underset{b=1}{\sum }}{\overset{ˇ}{\beta }}_{j}^{b}-{\overset{ˇ}{\beta }}_{j}\right)}}+\underset{\text{III}}{\underset{︸}{\sqrt{n}\left(\frac{1}{B}\overset{B}{\underset{b=1}{\sum }}\left\{{\tilde{\beta }}_{j}^{b}-{\overset{ˇ}{\beta }}_{j}^{b}\right\}\right)}}.$$

The first two terms in (12), which do not involve various selections *S^b^*’s, deal with the oracle estimators and the true active set *S**. We need to show the following, which will lead to the results stated in the theorem by using Slutsky’s theorem.

- $\text{I}/{\overset{ˇ}{\sigma }}_{j}=\sqrt{n}\left({\overset{ˇ}{\beta }}_{j}-\beta_{j}^{*}\right)/{\overset{ˇ}{\sigma }}_{j}\overset{d}{\to }N\left(0,1\right);$
- $\text{II=}\frac{\sqrt{n}}{B}\sum_{b=1}^{B}\left\{{\overset{ˇ}{\beta }}_{j}^{b}-{\overset{ˇ}{\beta }}_{j}\right\}=o_{p}\left(1\right);$
- $\text{III=}\frac{\sqrt{n}}{B}\sum_{b=1}^{B}\left\{{\tilde{\beta }}_{j}^{b}-{\overset{ˇ}{\beta }}_{j}^{b}\right\}=o_{p}\left(1\right)$.

First, (a) holds because of (11). To show (b), i.e. II = *o_p_*(1), we first denote $\xi_{b,n}=\sqrt{n}\left({\overset{ˇ}{\beta }}_{j}^{b}-{\overset{ˇ}{\beta }}_{j}\right)$, then $\text{II=}\left(\sum_{b=1}^{B}\xi_{b,b}\right)/B$. Since the sampling indicator vectors, **J**_*b*_’s (defined in Section 4) are i.i.d, *ξ_b,n_*’s are i.i.d conditional on data **D**^(*n*)^ = (**Y**, **X**). The conditional distribution of $\sqrt{n}\left({\overset{ˇ}{\beta }}_{j}^{b}-{\overset{ˇ}{\beta }}_{j}\right)$ given **D**^(*n*)^ is asymptotically the same as the unconditional distribution of $\sqrt{n}\left({\overset{ˇ}{\beta }}_{j}-\beta_{j}^{*}\right)$, which converges to zero Gaussian by (11). With the uniform boundedness of ${\overset{ˇ}{\beta }}_{j}^{b}$ and ${\overset{ˇ}{\beta }}_{j}$ as shown in Lemma 4, we can show that **E**(*ξ_b,n_*|**D**^(*n*)^) → 0 and $\text{Var}\left(\xi_{b,n}|D^{\left(n\right)}\right)\to {\overset{ˇ}{\sigma }}_{j}^{2}$ uniformly over **D**^(*n*)^ as *n* → ∞. Furthermore, **E**(II|**D**^(*n*)^) = **E**(*ξ_b,n_*|**D**^(*n*)^), and Var(II|**D**^(*n*)^) = Var(*ξ_b,n_*|**D**^(*n*)^)/*B*. Denote by Ω_*n*_ the sample space of **D**^(*n*)^. For any *δ, ζ* > 0, there exist *N*_0_, *B*_0_ > 0 such that when *n* > *N*_0_, *B* > *B*_0_,

$$\text{P}\left(\left|\text{II}\right|\geq \delta \right)\leq \int_{\Omega_{n}}P\left(\left|\text{II}\right|\geq \delta |D^{\left(n\right)}\right)\text{dP}\left(D^{\left(n\right)}\right)\;\leq \int_{\Omega_{n}}\text{P}\left(\left|\mathrm{II}-E\left(\text{II}|D^{\left(n\right)}\right)\right|\geq \delta /2|D^{\left(n\right)}\right)\text{dP}\left(D^{\left(n\right)}\right)\;\leq \int_{\Omega_{n}}\frac{\text{Var}\left(\text{II}|D^{\left(n\right)}\right)}{\delta^{2}/4}\text{dP}\left(D^{\left(n\right)}\right)\leq \frac{{\overset{ˇ}{\sigma }}_{j}^{2}}{B_{0}\delta^{2}/4}\int_{\Omega_{n}}\text{dP}\left(D^{\left(n\right)}\right)\leq \zeta$$

Thus, II = *o_p_*(1).

To prove (c), i.e. III = *o_p_*(1), we first note that each subsample $D_{1}^{b}$ can be regarded as a random sample of *n*_1_ = *qn* (0 < *q* < 1) i.i.d. observations from the population distribution for which Assumption (A3) holds, that is |*S^b^*| ≤ *K*_1_*n*^*c*1^ and P(*S** ⊆ *S^b^*) ≥ 1−*K*_2_(*p*∨*n*)^−1−*c*_2_^. We show that for any *b*, conditional on *S^b^* ⊇ *S**, $\sqrt{n}\left({\tilde{\beta }}_{j}^{b}-{\hat{\beta }}_{j}^{b}\right)=o_{p}\left(1\right)$.

To see this, we first arrange the order of the components of **x** = (*x*_1_, …, *x_p_*) such that the first s_0_ components are signal variables. In other words, *S** = {1, …, *s*_0_}. From (10) in in the proof of Theorem 1 and omitting superscript *b*, we have that

(13) $${\tilde{\beta }}_{j}-\beta_{j}^{*}=-n_{1}^{-1}{\tilde{e}}_{j}^{T}{\left\{I_{S_{+j}}^{*}\right\}}^{-1}U_{S_{+j}}\left(\beta_{S_{+j}}^{*}\right)+{\tilde{r}}_{n_{1}},\;{\overset{ˇ}{\beta }}_{j}-\beta_{j}^{*}=-n_{1}^{-1}{\overset{ˇ}{e}}_{j}^{T}{\left\{I_{S_{+j}^{*}}^{*}\right\}}^{-1}U_{S_{+j}^{*}}\left(\beta_{S_{+j}^{*}}^{*}\right)+{\overset{ˇ}{r}}_{n_{1}},$$

where ${\tilde{e}}_{j}=(0,\ldots ,0,1,0,\ldots ,0{)}^{T}$ is a unit vector of length |*S*_+*j*_| to index the position of variable *j* in *S*_+*j*_, ${\overset{ˇ}{e}}_{j}$ is a unit vector of length $\left|S_{+j}^{*}\right|$ to index the position of variable *j* in $S_{+j}^{*}$, and the residuals ${\left\|{\tilde{r}}_{n_{1}}\right\|}_{2}^{2}=o_{p}\left(1/n_{1}\right)$, ${\left\|{\overset{ˇ}{r}}_{n_{1}}\right\|}_{2}^{2}=o_{p}\left(1/n_{1}\right)$. Here, $I_{S_{+j}}^{*}$ and $I_{S_{+j}^{*}}^{*}$ are two submatrices of the expected information at ***β****, i.e. $I^{*}=E\left\{\frac{1}{n}{\bar{X}}^{\text{T}}V\bar{X}\right\}$, where **V** = diag{*ν*(*μ*_1_), …, *ν*(*μ_n_*)} is an *n* × *n* diagonal matrix with $\mu_{i}=g^{-1}\left({\bar{x}}_{i}\beta^{*}\right)$; see Section 2.1 for the notation.

Therefore, the *jk*-th (*j, k* = 0, 1, …, *p*) entry of *I**, a (*p* + 1) × (*p* + 1) matrix, is $E\left(x_{j}\nu \left(\mu \right)x_{k}\right)$ with $\mu =g^{-1}\left(\bar{\text{x}}\beta^{*}\right)$. Now for any *j* ∈ *S**, *k* ∈ *S**, the complement of *S**, the partial orthogonality condition (Fan and Lv, 2008; Fan and Song, 2010) that {*x_j_*, *j* ∈ *S**} are independent of {*x_k_, k* ∈ *S^c^*} implies that **E**(*x_j_ν*(*μ*)*x_k_*) = 0, as *μ* only depends on *x*′_*j*_, *j*′ ∈ *S** and **E**(*x_k_*) = 0 with centered predictors. Therefore, *I** is block-diagonal with two blocks indexed by *S** and *S^c^*. That is,

$$I^{*}=\left(\begin{array}{cc} E\left(\frac{1}{n}{\bar{X}}_{S^{*}}^{\text{T}}{V\bar{X}}_{S^{*}}\right) & 0\\ 0 & E\left(\frac{1}{n}X_{{\text{S}}^{c}}^{\text{T}}{\mathrm{VX}}_{S^{c}}\right) \end{array}\right).$$

where the submatrices ${\bar{X}}_{S^{*}}$ and **X**_*S^c^*_ are as defined in Section 2.1. Hence, $I_{S_{+j}}^{*}$ is block-diagonal with two blocks indexed by *S** and *S*_+*j*_\*S**, and $I_{S_{+j}^{*}}^{*}$ is block-diagonal with two blocks indexed by *S** and $S_{+j}^{*}\backslash S^{*}=0$ if *j* ∈ *S** or = {*j*} otherwise.

Therefore, {*I**}^−1^, ${\left\{I_{S_{+j}}^{*}\right\}}^{-1}$ and ${\left\{I_{S_{+j}^{*}}^{*}\right\}}^{-1}$ are all block-diagonal. Furthermore, the, blocks corresponding to *S** in ${\left\{I_{S_{+j}}^{*}\right\}}^{-1}$ and ${\left\{I_{S_{+j}^{*}}^{*}\right\}}^{-1}$ are identical and are equal to ${\left\{E\left(\frac{1}{n}{\bar{X}}_{S^{*}}^{T}{\text{V}\bar{X}}_{S^{*}}\right)\right\}}^{-1}$. Write $U\left(\beta^{*}\right)={\left(u_{0},u_{1},u_{2},\ldots ,u_{p}\right)}^{T}$, ${\tilde{e}}_{j}^{\text{T}}{\left\{I_{S_{+j}}^{*}\right\}}^{-1}={\left({\tilde{i}}_{jk}\right)}_{k\in S_{+j}}$ and ${\overset{ˇ}{e}}_{j}^{\text{T}}{\left\{I_{S_{+j}^{*}}^{*}\right\}}^{-1}={\left({\overset{ˇ}{i}}_{jk}\right)}_{k\in S_{+j}^{*}}$. Then, it follows that ${\tilde{i}}_{jk}={\overset{ˇ}{i}}_{jk}$ for *k* ∈ *S**, which leads to

$$\sqrt{n_{1}}\left({\tilde{\beta }}_{j}-{\overset{ˇ}{\beta }}_{j}\right)=-\frac{1}{\sqrt{n_{1}}}\underset{k\in S_{+j}}{\sum }{\tilde{i}}_{jk}u_{k}+\frac{1}{\sqrt{n_{1}}}\underset{k\in S_{+j}^{*}}{\sum }{\overset{ˇ}{i}}_{jk}u_{k}+{r^{\prime }}_{n_{1}}\;=-\frac{1}{\sqrt{n_{1}}}\underset{k\in S\backslash S^{*}}{\sum }{\tilde{i}}_{jk}u_{k}+\frac{1\left(j\notin S^{*}\right)}{\sqrt{n_{1}}}\left({\overset{ˇ}{i}}_{jj}-{\tilde{i}}_{jj}\right)u_{j}+{r^{\prime }}_{n_{1}}$$

where ${r^{\prime }}_{n_{1}}=\sqrt{n_{1}}\left({\tilde{r}}_{n_{1}}-{r^{\prime }}_{n_{1}}\right)=o_{p}\left(1\right)$, and ${\tilde{r}}_{n_{1}}$ and ${\overset{ˇ}{r}}_{n_{1}}$ are as in (13).

With Assumption (A3), $\left|S\backslash S^{*}\right|\leq K_{1}n^{c_{1}}=o\left(\sqrt{n_{1}}\right)$ with 0 ≤ *c*_1_ < 1/2 and, thus, $\text{Var}\left(\sum_{k\in S\backslash S^{*}}{\tilde{i}}_{jk}u_{k}\right)=o_{p}\left(1\right)$. By the Chebyshev inequality, the first term on the right hand side converges to 0 in probability. Thus, each of these three terms is *o_p_*(1) and we have $\sqrt{n_{1}}\left({\tilde{\beta }}_{j}-{\overset{ˇ}{\beta }}_{j}\right)=o_{p}\left(1\right)$. As *n*_1_/*n* = *q* where 0 < *q* < 1, the original statement holds.

Now define $\eta_{b}=1\left\{S^{*}⊈S^{b}\right\}\sqrt{n}\left({\tilde{\beta }}_{j}^{b}-{\overset{ˇ}{\beta }}_{j}^{b}\right)$, while omitting subscripts *j* in *η* for simplicity, then $\text{III=}\left(\sum_{b=1}^{B}\eta_{b}\right)/B$. When *S** ⊈ *S^b^*, ${\tilde{\beta }}_{j}^{b}$ is not an unbiased estimator of $\beta_{j}^{*}$ any more, instead we try to bound it by some constant. By Lemma 4, there exists *C_β_* ≥ *c_β_* such that ${\text{sub}}_{b}\left|{\tilde{\beta }}_{j}^{b}-{\overset{ˇ}{\beta }}_{j}^{b}\right|\leq {\text{sub}}_{b}\left|{\tilde{\beta }}_{j}^{b}-\beta_{j}^{*}\right|+{\text{sub}}_{b}\left|{\overset{ˇ}{\beta }}_{j}^{b}-\beta_{j}^{*}\right|\leq 2C_{\beta }+1$ Therefore, by (A3),

(14) $$E\left(\eta_{b}\right)\leq P\left(S^{*}⊈S^{b}\right)\sqrt{n}\underset{1\leq b\leq B}{\text{sup}}\left|{\tilde{\beta }}_{j}^{b}-{\overset{ˇ}{\beta }}_{j}^{b}\right|\leq 2C_{\beta }\sqrt{n}K_{2}{\left(p\vee n\right)}^{-1-c_{2}},\;\text{Var}\left(\eta_{b}\right)\leq \text{P}\left(S^{*}⊈S^{b}\right)n\underset{1\leq b\leq B}{\sup}{\left({\tilde{\beta }}_{j}^{b}-{\overset{ˇ}{\beta }}_{j}^{b}\right)}^{2}\leq 4C_{\beta }^{2}nK_{2}{\left(p\vee n\right)}^{-1-c_{2}}.$$

With dependent *η_b_*’s, we further have

$$E\left(\text{III}\right)=E\left\{\left(\overset{B}{\underset{b=1}{\sum }}\eta_{b}\right)/B\right\}\leq 2C_{\beta }\sqrt{n}K_{2}{\left(p\vee n\right)}^{-1-c_{2}},$$

$$\text{Var}\left(\text{III}\right)\leq \frac{1}{B^{2}}\overset{B}{\underset{b=1}{\sum }}\overset{B}{\underset{b^{\prime }=1}{\sum }}\left|\text{Cov}\left(\eta_{b},\eta_{b^{\prime }}\right)\right|\leq 4C_{\beta }^{2}nK_{2}{\left(p\vee n\right)}^{-1-c_{2}}$$

where the last inequality holds because of |Cov(*η_b_, η_b′_*)| ≤ {Var(*η_b_*)Var(*η_b′_*)}^1/2^ and (14). Then we show III = *o_p_*(1). More specifically, for any *δ* > 0, *ζ* > 0, take *N*_0_ = ⌊(*C_β_δ*)^1/2+*c*_2_^⌋, where, for a real number *a*, ⌊*a*⌋ denotes the integer part of *a*. When *n* > *N*_0_, **E**(III) ≤ *δ*/2. Also let $N_{1}=\left\lfloor {\left\{\zeta \delta^{2}/\left(16C_{\beta }^{2}K_{2}\right)\right\}}^{c_{2}}\right\rfloor$. Then when *n* > max(*N*_0_, *N*_1_), we have

$$\text{P}\left(\left|\text{III}\right|\geq \delta \right)\leq \text{P}\left(\left|\text{III}-E\left(\text{III}\right)\right|\geq \delta /2\right)\;\leq \frac{\text{Var}\left(\text{III}\right)}{\delta^{2}/4}\leq \frac{16C_{\beta }^{2}K_{2}}{\delta^{2}}n{\left(p\vee n\right)}^{-1-c_{2}}\;\leq \frac{16C_{\beta }^{2}K_{2}}{\delta^{2}}n^{-c_{2}}<\zeta ,$$

where the first inequality is due to |**E**(III)| ≤ *δ*/2 when *n* > *N*_0_, the second one is due to the Chebyshev inequality and the last one is due to *n* > *N*_1_. ■

**Proof** of Theorem 3:

Following the previous proof, we replace the arguments in *j* with those in *S*^(1)^. The *oracle* estimators are

$${\overset{ˇ}{\beta }}_{S^{\left(1\right)}\cup S^{*}}=\text{argmin}\ell_{S^{\left(1\right)}\cup S^{*}}\left(\beta_{S^{\left(1\right)}\cup S^{*}};Y,X_{S^{\left(1\right)}\cup S^{*}}\right),{\overset{ˇ}{\beta }}_{S^{\left(1\right)}}={\left({\overset{ˇ}{\beta }}_{S^{\left(1\right)}\cup S^{*}}\right)}_{S^{\left(1\right)}};$$

$${\overset{ˇ}{\beta }}_{S^{\left(1\right)}\cup S^{*}}^{b}=\text{argmin}\ell_{S^{\left(1\right)}\cup S^{*}}\left(\beta_{S^{\left(1\right)}\cup S^{*}};Y^{1\left(b\right)},X_{S^{\left(1\right)}\cup S^{*}}^{1\left(b\right)}\right),{\overset{ˇ}{\beta }}_{S^{\left(1\right)}}^{b}={\left({\overset{ˇ}{\beta }}_{S^{\left(1\right)}\cup S^{*}}^{b}\right)}_{S^{\left(1\right)}}.$$

Notice that |*S*^(1)^| = *p*_1_ = *O*(1), as *n* → ∞, |*S** ∪ *S*^(1)^| = *O*(|*S**| = *o*(*n*)), so that the above quantities are well-defined. The oracle estimator follows

$$\sqrt{n}{\left\{{\left(I_{S^{\left(1\right)}\cup S^{*}}^{*}\right)}^{-1/2}\right\}}_{S^{\left(1\right)}}\left({\overset{ˇ}{\beta }}_{S^{\left(1\right)}}-\beta_{S^{\left(1\right)}}^{*}\right)\overset{d}{\to }N\left(0,{\text{I}}_{p1}\right)\;\text{as}\;n\to \infty .$$

Here, for a square matrix, say, *Q*, (*Q*)_*S*_ is a submatrix of *Q* with rows and columns indexed by *S*. Denote by $I^{\left(1\right)}={\left\{{\left(I_{S^{\left(1\right)}\cup S^{*}}^{*}\right)}^{-1/2}\right\}}_{S^{\left(1\right)}}$.

Similar to (12), we have a decomposition

$$\sqrt{n}I^{\left(1\right)}\left({\hat{\beta }}^{\left(1\right)}-\beta_{S^{\left(1\right)}}^{*}\right)=\sqrt{n}I^{\left(1\right)}\left({\overset{ˇ}{\beta }}_{S^{\left(1\right)}}-\beta_{S^{\left(1\right)}}^{*}\right)+\sqrt{n}I^{\left(1\right)}\left(\frac{1}{B}\overset{B}{\underset{b=1}{\sum }}{\tilde{\beta }}_{S^{\left(1\right)}}^{b}-{\overset{ˇ}{\beta }}_{S^{\left(1\right)}}\right).$$

Analogous to the derivations in the previous proof, it follows that the second term is *o_p_*(1). Hence, the theorem holds. ■

## Appendix B: Additional Simulations

To assess the robustness of our method, we performed additional simulations when the parametric model was mis-specified and when the sparsity condition was violated.

**Example B.1** assumed that *Y*|**x** followed a negative binomial distribution:

$$P\left(Y=y\right)=\frac{\Gamma \left(y+r\right)}{\Gamma \left(r\right)y!}p^{r}{\left(1-p\right)}^{y},$$

$$EY=\mu =r\left(1-p\right)/p=x\beta^{*},$$

with *r* = 10, sample size *n* = 300, *p* = 500, and *s*_0_ = 5. However, we modeled the data using SSGLM under the Poisson regression (8) with *B* = 300. Table 8 summarizes the results based on 200 simulated data sets. The ${\hat{\beta }}_{j}’\text{s}$ had small biases. The estimated standard errors were slightly less than the empirical standard deviations. Nevertheless, the coverage probabilities were still close to the 0.95 nominal level.

**Example B.2** assumed a non-sparse truth ***β**** under the Poisson truth (8). With *n* = 300 and *p* = 500, we let *s*_0_ = 100. Among the 100 predictors with non-zero effects, ${\hat{\beta }}_{j}’\text{s}$ were small, which were randomly drawn from Unif[−0.5, 0.5], and the other 4 had values −1.5, −1, 1, 1.5 (as shown in Figure 3). With many small but non-zero signals, SSGLM still gave nearly unbiased estimates to all of them. See Table 9, where the columns represent 4 large size ${\hat{\beta }}_{j}’\text{s}$, and the averages over all small signals and all noise variables, respectively.

**Table 8:** SSGLM under mis-specified model.

Index *j*	90	179	206	237	316	Noise
$\beta_{j}^{*}$	−1.000	−0.500	0.500	1.000	1.500	0.000

Bias	−0.020	0.020	0.018	0.001	0.010	−0.001
SE	0.240	0.235	0.232	0.236	0.243	0.233
SD	0.258	0.243	0.249	0.249	0.250	0.231
Cov prob	0.955	0.945	0.900	0.930	0.925	0.946
Sel freq	0.724	0.177	0.216	0.723	0.977	0.021

**Table 9:** SSGLM under non-sparse truth.

Index *j*	128	256	381	497	Small	Noise
$\beta_{j}^{*}$	−1.50	−1.00	1.00	1.50	-	0

Bias	−0.01	0.003	−0.03	0.05	0.003	9 × 10^−4^
SE	0.31	0.30	0.30	0.31	0.29	0.30
SD	0.31	0.30	0.32	0.30	0.29	0.29
Cov prob	0.93	0.93	0.93	0.94	0.94	0.94
Sel freq	0.87	0.50	0.49	0.87	0.06	0.03
